# Maize residue retention shapes soil microbial communities and co-occurrence networks upon freeze-thawing cycles

**DOI:** 10.7717/peerj.17543

**Published:** 2024-06-14

**Authors:** Yang Yu, Quankuan Guo, Shuhan Zhang, Yupeng Guan, Nana Jiang, Yang Zhang, Rong Mao, Keyu Bai, Salimjan Buriyev, Nuriddin Samatov, Ximei Zhang, Wei Yang

**Affiliations:** 1 College of Resources and Environment, Northeast Agricultural University, Harbin, China; 2 Institute of Environment and Sustainable Development in Agriculture, Chinese Academy of Agricultural Sciences, Beijing, China; 3 College of Forestry, Jiangxi Agricultural University, Nanchang, China; 4 Institute of Agricultural Resources and Regional Planning, Chinese Academy of Agricultural Sciences, Beijing, China; 5 Institute of Environment and Nature Conservation Technologies of the Ministry of Ecology, Environmental Protection, and Climate Change of the Republic of Uzbekistan, Tashkent, Uzbekistan

**Keywords:** Maize residue retention, Freezing-thawing cycles, Bacteria, Fungi, Soil quality, Co-occurrence network

## Abstract

Maize residue retention is an effective agricultural practice for improving soil fertility in black soil region, where suffered from long freezing-thawing periods and intense freeze-thawing (FT) cycles. However, very few studies have examined the influence of maize residue retention on soil microbial communities under FT cycles. We investigated the response of soil microbial communities and co-occurrence networks to maize residue retention at different FT intensities over 12 cycles using a microcosm experiment conditioned in a temperature incubator. Our results indicated that maize residue retention induced dramatic shifts in soil archaeal, bacterial and fungal communities towards copiotroph-dominated communities. Maize residue retention consistently reduced soil fungal richness across all cycles, but this effect was weaker for archaea and bacteria. Normalized stochastic ratio analysis revealed that maize residue retention significantly enhanced the deterministic process of archaeal, bacterial and fungal communities. Although FT intensity significantly impacted soil respiration, it did not induce profound changes in soil microbial diversity and community composition. Co-occurrence network analysis revealed that maize residue retention simplified prokaryotic network, while did not impact fungal network complexity. The network robustness index suggested that maize residue retention enhanced the fungal network stability, but reduced prokaryotic network stability. Moreover, the fungal network in severe FT treatment harbored the most abundant keystone taxa, mainly being cold-adapted fungi. By identifying modules in networks, we observed that prokaryotic Module #1 and fungal Module #3 were enhanced by maize residue retention and contributed greatly to soil quality. Together, our results showed that maize residue retention exerted stronger influence on soil microbial communities and co-occurrence network patterns than FT intensity and highlighted the potential of microbial interactions in improving soil functionality.

## Introduction

Black soil (classified as Mollisol), which is characterized by a black and thick humic topsoil layer, is of high fertility and very suitable for crop growth. As one of the most precious soil resources in China, black soil is faced with serious soil erosion and fertility deterioration over the past several decades (Liu & Diamond, 2005; Yao et al., 2017). Maize residue retention is an advocated agricultural practice for improving soil fertility and crop yield in black soil region (Gu et al., 2018; Guan et al., 2022). It enables the utilization of straw resources while effectively ameliorate soil water use efficiency, prevent soil erosion and enhance soil fertility (Guan et al., 2022; Liu et al., 2021; Zhanget al., 2021). Moreover, the benefits of maize residue retention on soil fertility were also reflected at its effect on soil microorganisms (Wu et al., 2023).

Soil microorganisms are crucial component of soil ecosystem and contribute greatly to the process of straw decomposition (Fierer, 2017; Yang et al., 2021). Maize residue retention provides large amounts of substrate for soil microbes and improves soil nutrient availabilities, and thereby may enhance soil microbial biomass, activity and diversity (Yao et al., 2017). However, the effects of maize residue retention on soil microorganisms would depend on various factors including the climate, application time and types of straw. In cold regions, where low temperature and frequent freeze-thawing (FT) cycles are limiting factors for the crop residue decomposition, there is still uncertainty of maize residue retention on soil microbial communities (Gu et al., 2020; Guan et al., 2022).

FT is a common phenomenon in black soil region during winter (Groffman et al., 2010; Wei et al., 2016), and it encompasses two physical processes: soil freezing and melting. Previous microcosm and field studies have shown that FT cycles would impose complex effects on soil microbial communities in several ways (Haei et al., 2011; Han et al., 2018; Yanai, Toyota & Okazaki, 2011). Firstly, FT may directly disrupt soil microbial communities through lysis of microbial cells due to ice crystal formation (Yanai, Toyota & Okazaki, 2011), and 7% of soil microorganisms may die by repeated FT (Ji & Wang, 2022). Secondly, the releasing nutrients from dead microbial cells and disruption of aggregates together lead to a rapid increase in soil available nutrients, which trigger the growth of soil microbes and induce changes in their community composition after thawing (Haei et al., 2011; Han et al., 2018). These changes may further influence soil enzyme activities, as well as the straw decomposition process. Consequently, understanding how soil microbial communities respond to FT would offer a more comprehensive insight into the performance of maize residue retention in cold regions.

In agricultural soils, the myriad of microbes lives together and form complex interconnected microbial networks, where microbes associate with each other directly or indirectly through processes, such as competition, predation, and mutualism (de Vries et al., 2018; Wagg et al., 2019). It is theoretically expected that microbial communities with more complex associations will have more active metabolic processes and faster growth rates, resulting in improved community performance (Brown et al., 2004; Chen et al., 2023; Jordan, 2009). Previous researchers have tried to link microbial network complexity to ecosystem multifunctionality (Chen et al., 2022; Wagg et al., 2019), and Chen et al. (2022) reported that soil microbial network complexity contributed more to multifunctionality than diversity. Therefore, elucidating the complexity and stability of these microbial associations based on network analysis would provide more meaningful information than community analysis (Deng et al., 2012; Yuan et al., 2021). In recent years, a few studies have reported the effect of organic input (*e.g.*, compost, crop residue) on the microbial co-occurrence network patterns. For instance, Xu et al. (2023) reported that maize residue retention complicates and stabilizes the soil microbial networks. However, the effects of FT on soil microbial networks are far less understood than that of residue retention, especially lacking the interactive effects of maize residue retention and FT. More importantly, very little is known of whether differences in the microbial networks have consequences for microbiome function upon maize residue retention.

Sanjiang Plain is located in the seasonal frozen soil area in Northeast China, suffering from long freeze-thaw periods and intense freeze-thaw cycles (Ouyang et al., 2013). Maize residue retention is an advocated agricultural practice to increase the contents of soil available nutrients in this region (Shen et al., 2018), and it will be crucial to emphasize the interactive effect of maize residue retention and FT on the soil microbial communities. Therefore, we conducted a microcosm experiment to examine the response of soil quality, microbial diversity, community composition, co-occurrence network to residue retention and FT. We hypothesized: (1) Soil microbial communities and co-occurrence networks would be affected by maize residue retention and FT; (2) Maize residue retention would exert a stronger effect on soil microbes than FT; (3) Maize residue retention would improve soil quality, and this effect would be mediated through soil microbial network properties.

## Materials and Methods

### Soil collection and experimental design

The study was conducted at Institute of Environment and Sustainable Development in Agriculture, Chinese Academy of Agricultural Sciences, Beijing in 2022. The experimental design was a fully-factorial experimental design with three factors. One factor is maize residue retention, containing two treatments: no maize residue retention (control, CK), maize residue retention (RR). To stimulate the maize residue retention in field, the amount of maize residue incorporated was approximately equal to the application rate (13th m^−2^) in Sanjiang Plain. The second factor is FT intensity, containing three treatments: constant at 4 °C (no FT), −4 °C/4 °C (moderate FT), and −10 °C/4 °C (severe FT). The soil was frozen at −4 °C or −10 °C for 2 h and then thawed at 4 °C within 12 h, repeat twice, which was regarded as a freeze–thawing cycle. The third factor is the number of FT cycles. In black soil region, soils generally experience 7–12 FT cycles (Liu et al., 2024), so the FT cycles were set to one, three, six, and 12 cycles. Each treatment was replicated four times, resulting in 96 pots (capacity: 4 cm in diameter, 7 cm in height) in total.

Soils used in the present study was collected from maize cropland in Sanjiang Plain (47°12′41.04″N, 130°24′4.68″E) in August 30th, 2022. The soil at this study site is classified as Mollisol (Soil Survey Staff, 2022). In brief, 10 plots (1 m × 1 m) were randomly selected in the sampling sites. Then soil samples (0∼20 cm) were collected using a spade and mixed thoroughly. The fresh maize stalks (water content: 92.5%) were collected in the same year, and dried to constant weight. Then the dry maize stalks were cut into pieces of approximately one cm length manually. Soil samples were evenly mixed or not mixed with maize straw after sieved through two mm mesh, and incubated at 25 °C for 30 days. We added approximately 200 g soils and 5 g of maize straw stalks in each pot, and placed all pots in incubators. After each cycle, the pots were taken out without being put back, the filed water holding capacity was maintained at 24% during the period. Soils in each pot were divided into three parts, and stored at 4 °C, −80 °C and room temperature, respectively.

### Soil physiochemical variables and enzyme activities determination

The soil moisture content was measured with fresh soil by the drying method (Bao, 2000). Soil pH was measured with dry soil by potentiometer according to the ratio of soil to water 2.5:1 (Bao, 2000). Soil total carbon (TC) and nitrogen (TN) were determined with dry soil by elemental analyser (Elementar-vario EL cube, Germany). Soil nitrate N (NO_3_^−^-N) and ammonia N (NH_4_^+^-N) were determined with fresh soil by flow analyzer (Seal-AA3, Germany). The available phosphorus (AP) was determined with dry soil by the molybdenum-antimony resistance colorimetric method. The available potassium (AK) was determined with dry soil by flame atomic absorption spectrometry (Yang et al., 2019). Soil microbial respiration was determined with fresh soil by LI-850 CO_2_/H_2_O analyzer (LI-CON, US) (Fujita, Noguchi & Terashima, 2013).

In this study, six soil enzymes related to C, N, and P metabolism were selected, including β-D-glucosidase (BG), β-D-xylosidase (XYL), urease (UE), leucine aminopeptidase (LAP), polyphenol oxidase (PPO) and acid phosphatase (APE) activities. The BG, XYL, LAP and APE activities were measured fluorometrically using MUB-linked model substrates (Fernley & Walker, 1965; Saiya-Cork, Sinsabaugh & Zak, 2002) with a microplate flurometer (Tecan, Infinite 200 PRO). The PPO and UE activities were measured using spectrophotometry method, and soil polyphenol oxidase (S-PPO) activity test kit (Solebol Reagent Company, item No.: BC0110) and soil urease (S-UE) activity test kit (Solebol Reagent Company, item No.: BC0110) were used respectively (DeForest, 2009).

### DNA extraction and Miseq sequencing

Total DNA was extracted from 0.25 g fresh soil using PowerSoil DNA isolation kit (Qiagen, Hilden, Germany). Prokaryotic 16S rDNA region was amplified with primer 515F (5′-GTGCCAGCMGCCGCGGTAA-3′) and 806R (5′-GGACTACHVGGGTWTCTAAT-3′) (Wasimuddin et al., 2020), and fungal ITS2 fragment was amplified with primer gITS7 (5′-GTGARTCATCGARTCTTTG-3′) and ITS4 (5′-TCCTCCGCTTATTGATATGC-3′) (Ihrmark et al., 2012). Primer 515F and gITS7 contained a 12 bp barcode unique to each sample for Miseq sequencing detection. All PCR reactions followed (Guan et al., 2022) within a 25 mL reaction system. The PCR products were detected by electrophoresis and purified. The DNA concentration of purified PCR product was determined using Nanodrop2000 (Thermoscientific, USA), 50 ng DNA was taken from each DNA sample and corrected to 10 ng µL^−1^. The corrected samples were then sequenced using Illumina Miseq platform at Majorbio Biotech Co., Ltd. (Shanghai, China). The raw sequence data have been deposited on the NCBI SRA, with accession number PRJNA1045363 (SRR26950863 –SRR26951054).

### Raw sequence processing and taxonomic classification

Quantitative insight into microbial ecology (QIIME) PipelineVersion1.8.0 (Caporaso et al., 2010) was used to remove sequences that contained incorrect primers, fuzzy bases, the same continuous base >8 or average quality values <25. The “chimera.uchime” command in Mothur software was used to remove potential chimera sequences. Prokaryotic sequences were then error-filtered and grouped into amplicon sequence variants (ASVs) using the Deblur software (Amir et al., 2017). The ASVs were blasted against the silva 16s database and UNITE database to annotate their taxonomy, and ASVs that are not assigned as prokaryotes or fungi were removed. The number of sequences per sample was rarefied to 32,582 and 11,975 for prokaryotes and fungi using the “vegan” package (version: 2.6–4, Oksanen et al., 2020), respectively. Furthermore, the archaeal and bacterial ASVs were picked from prokaryotic ASV tables and rarefied, respectively.

### Data analysis

All of the following analysis were conducted using R 3.6.0 (R Core Project, 2023). Archaeal, bacterial and fungal diversity indices were calculated for each treatment using the “vegan” software package. Fungal trophic modes (pathotroph, saprotroph, symbiotroph) were annotated using FUNGuild (Nguyen et al., 2016). The functional profiles of bacteria were predicted using the FAPROTAX. The effects of maize residue retention and FT intensity on soil physicochemical properties, enzyme activities, respiration, and prokaryotic/fungal diversity and fungal trophic modes were analyzed by mixed effect model (random =∼1|Block/Plot, correlation = corCAR1(form =∼FT cycle|block/plot) using the “nlme” package (Pinheiro et al., 2013). All data were tested for normality and homogeneity of variance. The prokaryotic and fungal community compositions were ordinated by principal co-ordinate analysis (PCoA) based on bray-curtis dissimilarity with “vegan” package (Oksanen et al., 2020). Then the effect of maize residue retention, FT intensity and FT cycles were examined using permanova analysis with 999 permutations by using “vegan” package (Oksanen et al., 2020). Mantel test in “ecodist” software package (Goslee & Urban, 2020) was used to analyze the relationship between soil prokaryotic/fungal community composition and soil physiochemical properties. The normalized stochasticity ratio (NST) was calculated to examine the community assembly process es of archaea, bacteria and fungi using the “NST” package (Ning et al., 2019).

Co-occurrence networks were constructed for soil prokaryotic and fungi based on all soil samples using the package “igraph” (Csardi & Nepusz, 2006). ASVs with relative frequency >50% are retained for network construction. The Spearman correlation coefficient among different ASVs was calculated using the “psych” software package. After the *P* value was corrected by FDR, the correlations with *P* > 0.01 and *r* < 0.6 were removed. Nodes with a value of among-module connectivity >0.625 or within-module connectivity >2.5 are identified as keystone species (Guimerà & Amaral, 2005). Module, which is a group of nodes that densely connected to each other than to nodes outside the group, were identified using “igraph” package. The network topological properties including edges, connectedness and robustness were calculated. We also constructed sub-networks for each treatment to compare the different network patterns.

Soil quality index is a synthetic parameter calculated from the average value of *z*-score transformation of APE, BG, XYL, LAP, PPO, UE, AK, AP, NH_4_^+^-N, NO_3_^−^-N, soil respiration, TN and TC, which could reflect the soil function comprehensively. The random forest model was used to explore the contribution of soil microbial diversity indices, network modules, topological properties and the positive edges/negative edges (P/N) ratio to soil quality.

## Results

### Soil physiochemical properties, enzyme activities and respiration

Mixed effect model revealed that soil nutrient availabilities (including available potassium (AK), available phosphorus (AP), total nitrogen (TN)) and enzyme activities (β-D-glucosidase (BG), β-D-xylosidase (XYL), leucine aminopeptidase (LAP), polyphenol oxidase (PPO)) were significantly affected by maize residue retention. Compared to control, soil TN, AK and AP were enhanced by 7.3%, 62.9% and 19.2% in residue retention treatment. Among the soil enzyme indices, BG, XYL, and LAP were enhanced by residue retention by 49.2%, 20.1% and 7.7%, respectively, while PPO was reduced by 14.4% in residue retention treatment (Table S1, all *P* < 0.05). Although FT intensity showed no effect on the variables mentioned above (Table S1, all *P* > 0.05), it significantly impacted soil respiration (*P* < 0.001). Moderate and severe FT intensity significantly reduced soil respiration in control, but did not impact soil respiration under maize residue retention (Fig. S1). In addition, soil AK, ammonia N (NH_4_^+^-N), total carbon (TC), XYL, LAP, PPO and respiration exhibited significant variations among FT cycles (Table 1). Soil pH, nitrate N (NO_3_^−^-N) and acid phosphatase (APE) were unaffected by residue retention, FT intensity and cycles.

**Table 1:** The mixed effect models with freeze-thawing (FT) cycles autocorrelation were used to evaluate the influence of maize residue retention (RR), FT, and interaction between RR and FT on each index. (random =∼1—Block/Plot, correlation = corCAR1(form =∼FT cycle—block/plot)). The data in the table are *P* values calculated from the mixed effect module.

Variables	FT cycle	RR	FT intensity	RR*FT intensity
AK (mg kg^−1^)	0.043	<0.001	0.588	0.346
AP (mg kg^−1^)	0.065	<0.001	0.974	0.667
pH	0.603	0.157	0.316	0.969
NH_4_^+^-N (mg kg^−1^)	0.036	0.135	0.603	0.091
NO_3_^−^-N (mg kg^−1^)	0.087	0.260	0.428	0.999
TN (%)	0.094	<0.001	0.214	0.738
TC (%)	<0.001	0.098	0.381	0.427
APE (nmol h^−1^ g^−1^)	0.332	0.138	0.610	0.690
BG (nmol h^−1^ g^−1^)	0.204	<0.001	0.247	0.580
XYL (nmol h^−1^ g^−1^)	<0.001	0.018	0.734	0.522
LAP (nmol h^−1^ g^−1^)	<0.001	<0.001	0.523	0.940
PPO (mg g^−1^ d^−1^)	<0.001	0.025	0.073	0.091
UE (mg g^−1^ d^−1^)	0.077	0.825	0.667	0.999
Respiration (µmol mol^−1^)	<0.001	0.151	0.015	0.011
MBC (mg kg^−1^)	0.054	<0.001	0.491	0.746
BAC_S	0.018	<0.001	0.231	0.882
FUN_S	0.002	<0.001	0.386	0.750
ARCH_S	<0.001	<0.001	0.162	0.316

**Notes.**

Abbreviations AK available potassium AP available phosphorus NO_3_^−^-N soil nitrate N NH_4_^+^-N soil ammonia N TC total carbon TN total nitrogen APE acid phosphatase BG β-D-glucosidase XYL β-D-xylosidase LAP leucine aminopeptidase PPO polyphenol oxidase UE urease MBC microbial biomass carbon BAC_S bacterial richness FUN_S fungal richness ARCH_S archaeal richness RR maize residue retention FT freeze-thawing RR*FT intensity the interaction between RR and FT intensity

*P* values: *P* > 0.05, not significant; *P* < 0.05, significant.

### Soil prokaryotic and fungal communities

**Figure 1:**
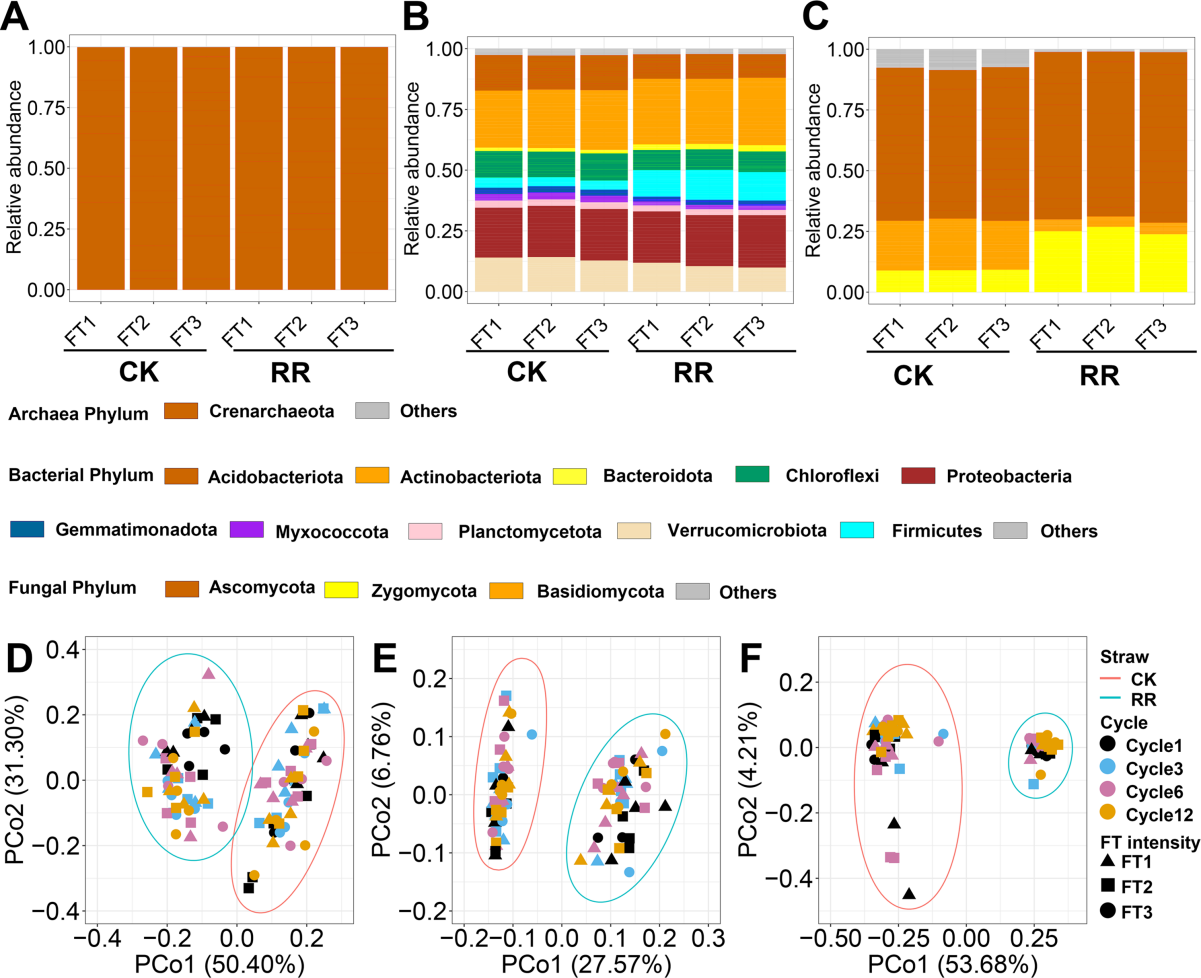
Relative abundance of archaeal (A), bacterial (B) and fungal (C) phyla. Principal coordinate analysis of soil archaeal (D), bacterial (E) and fungal (F) community compositions among treatments. Abbreviations: CK, control; RR, maize residue retention; Cycle, freeze-thawing cycles; FT, freeze-thawing; FT1, constant 4  °C; FT2, −4 °C/ 4° C (moderate FT intensity), FT3, −10 °C/ 4 °C (severe FT intensity). Cycle1, Cycle3, Cycle6 and Cycle12 represents for one, three, six and 12 freeze-thawing cycles, respectively.

A total of 32,582 prokaryotic ASVs and 11,975 fungal ASVs were obtained after quality control and flattening. Among prokaryotic ASVs, 32,355 ASVs belonged to bacteria, and 227 ASVs belonged to archaea. Crenarchaeota (99.9%) was the predominant phylum for archaea, while other phyla only occupied a minor fraction of archaeal communities (Fig. 1A). Actinobacteriota (25.6% in total abundance) was the dominant phylum for bacteria, followed by Proteobacteria (21.1%), Verrucomicrobiota (12.2%) and Acidobacteriota (12.2%) (Fig. 1B). For fungi, Ascomycota (65.7%), Zygomycota (17.1%) and Basidiomycota (12.7%) dominate their communities and occupied 95.49% of the total abundance (Fig. 1C).

**Figure 2:**
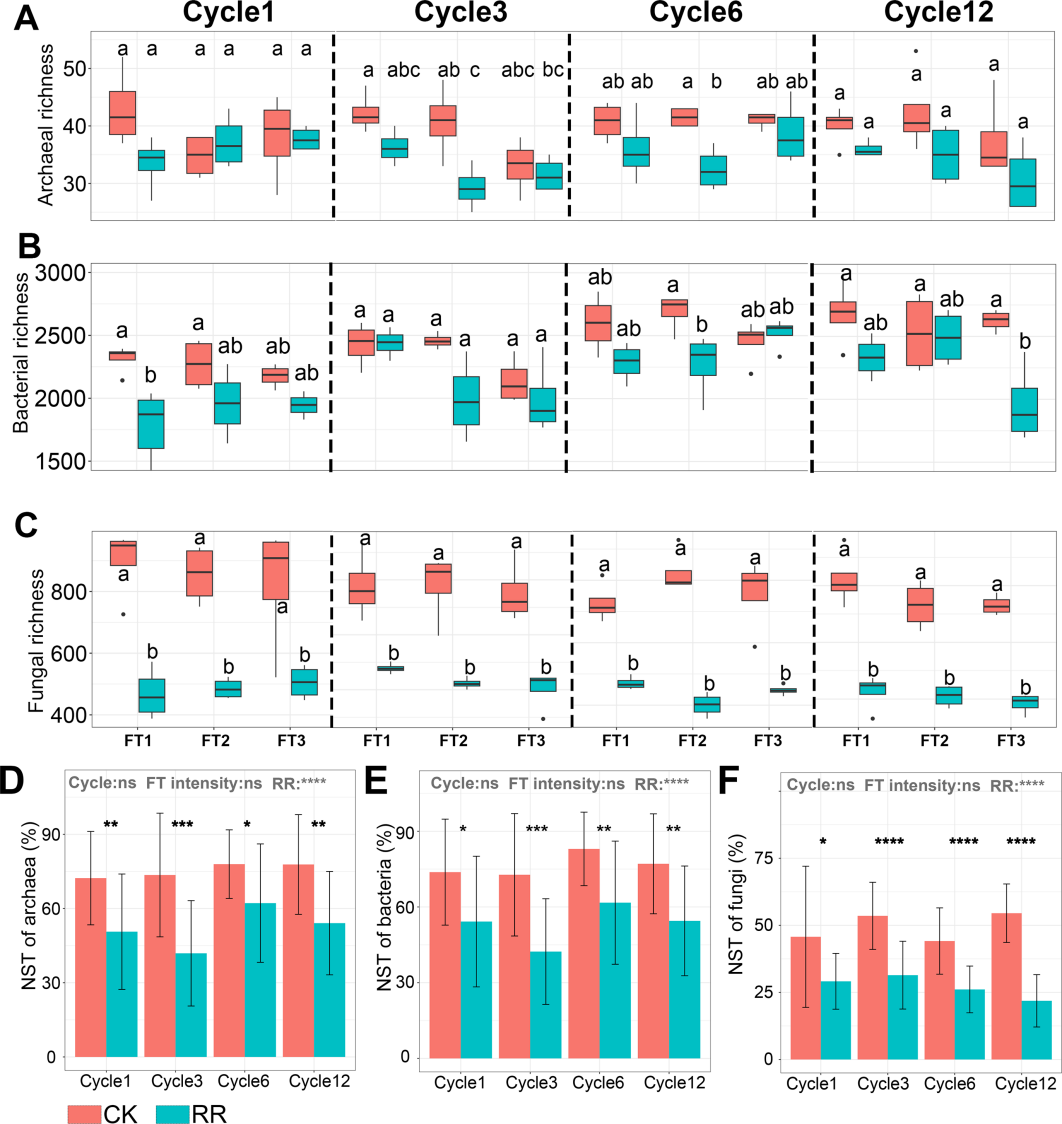
Box plots showing the archaeal (A), bacterial (B) and fungal (C) richness among treatments in Cycle1, Cycle3, Cycle6 and Cycle12. Bar plots showing the normalized stochastic ratio of archaeal (D), bacterial (E) and fungal (F) community assembly. Abbreviations: CK, control; RR, maize residue retention; Cycle, freeze-thawing cycles; FT, freeze-thawing; FT1, constant 4 °C; FT2, −4 °C/ 4 °C (moderate FT intensity), FT3, −10 °C/ 4 °C (severe FT intensity). Cycle1, Cycle3, Cycle6 and Cycle12 represents for one, three, six and 12 freeze-thawing cycles, respectively. In A-C, box plots without shared letters indicate significant difference at *P* < 0.05. In (D–F), symbols indicate the *P* values from t test: *, 0.01 < *P* < 0.5; **, 0.001 < *P* < 0.01; ***, 0.0001 < *P* < 0.001; **** *P* < 0.0001; ns, not significant.

Soil archaeal (Fig. 1D), bacterial (Fig. 1E) and fungal (Fig. 1F) community composition were ordinated using PCoA based on Bray-Curtis dissimilarity. The ordination plots clearly indicated that they were all separated by maize residue retention, which was also supported by permanova analysis (Table S2). Moreover, soil bacteria (*P* = 0.02) and fungal (*P* = 0.027) community compositions also exhibited obvious difference among FT cycles (Table S2). However, FT intensity did not significantly impact soil microbial community composition (Table S2). The shift of soil microbial communities was also reflected at the phylum level, with multiple phyla were enriched or depleted by residue retention. The relative abundance of Acidobacteriota, Chloroflexi, and Basidiomycota were reduced by 31.1% (Fig. 1B), 23.6% (Fig. 1B) and (77.4%, Fig. 1C) by residue retention, while Actinobacteriota, Firmicutes, and Zygomycota were enriched by 11.7% (Fig. 1B), 195.7% (Fig. 1B) and 179.2% (Fig. 1C), respectively. Maize residue retention consistently reduced soil fungal (Fig. 2C) richness across all cycles. However, the residue retention exhibited weaker effect on archaeal (Fig. 2A) and bacterial richness as compared with fungi, which only occasionally reduced archaeal/bacterial richness across 12 cycles (Table 1, Fig. 2B).

### Soil prokaryotic and fungal community assembly process

Normalized stochasticity ratio (NST) analysis revealed that both of archaeal (Fig. 2D) and bacterial (Fig. 2E) communities were dominated by stochastic process (the average NST value was 64.9% and 63.8%, respectively), and fungal (Fig. 2F) community was dominated by deterministic process (the average NST value was 38.3%). Maize residue retention significantly enhanced the deterministic process of archaeal (Fig. 2D), bacterial (Fig. 2E) and fungal (Fig. 2F) communities. Moreover, FT intensity and FT cycles did not impact the archaeal (Fig. 2D), bacterial (Fig. 2E) and fungal (Fig. 2F) community assembly process.

### Prokaryotic and fungal co-occurrence networks

**Figure 3:**
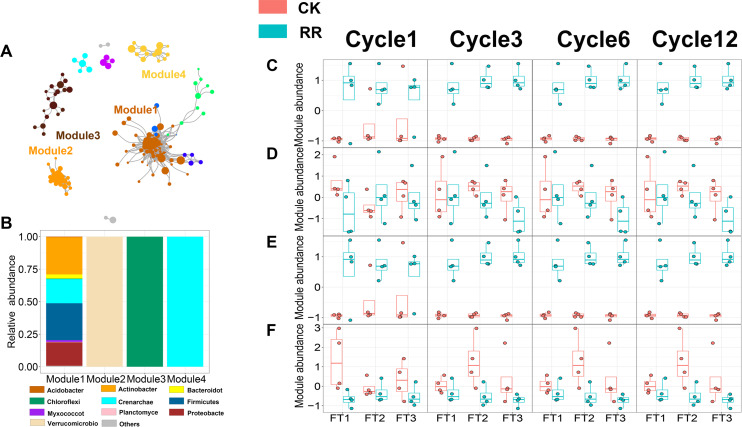
Soil prokaryotic (A) co-occurrence networks with nodes colored according to each of the four main modules. The relative abundance of the main prokaryotic (B) phyla in the four modules. The relative abundance (*z*-score) of Module #1, Module #2, Module #3 and Module #4 (prokaryotic modules: C, D, E and F) among treatments. Abbreviations: CK, control; RR, maize residue retention; Cycle, freeze-thawing cycles; FT, freeze-thawing; FT1, constant 4 °C; FT2, −4 °C/ 4° C (moderate FT intensity), FT3, −10 °C/ 4 °C (severe FT intensity). Cycle1, Cycle3, Cycle6 and Cycle12 represents for one, three, six and 12 freeze-thawing cycles, respectively.

**Figure 4:**
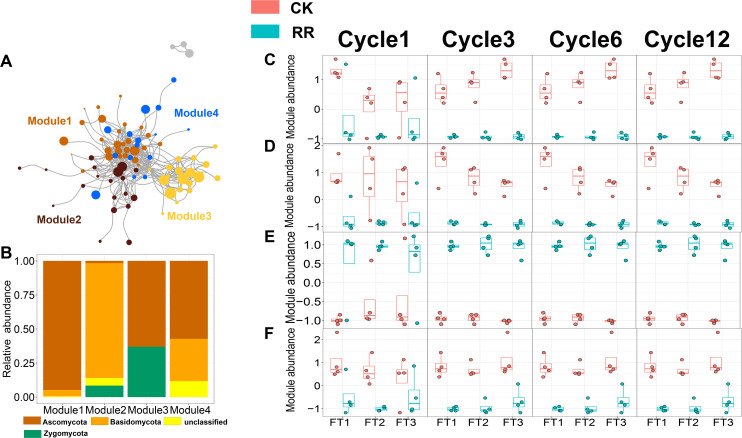
Soil fungal (A) co-occurrence networks with nodes colored according to each of the four main modules. The relative abundance of the main fungal (B) phyla in the four modules. The relative abundance (*z*-score) of Module #1, Module #2, Module #3 and Module #4 (fungal modules: C, D, E and F) among treatments. Abbreviations: CK, control; RR, maize residue retention; Cycle, freeze-thawing cycles; FT, freeze-thawing; FT1, constant 4 °C; FT2, −4 °C/4 °C (moderate FT intensity), FT3, −10 °C/4 °C (severe FT intensity). Cycle1, Cycle3, Cycle6 and Cycle12 represents for one, three, six and 12 freeze-thawing cycles, respectively.

As shown in Figs. 3 and 4, prokaryotic network was larger and more connected than fungal network. We then visualized modules with more than five nodes in networks, and focused on the top 4 modules for both prokaryotes and fungi. For prokaryotic network, Module #1 was the largest module and composed of multiple phyla, mainly including Actinobacteriota, Crenarchaeota, Proteobacteria and Firmicutes. Module #2, #3, and #4 were each composed of a single phylum: Verrucomicrobiota, Chloroflexi, and Crenarchaeota, respectively (Fig. 3B). The relative abundance of Module #1 in the maize residue retention treatment was significantly higher than in the control. Module #1 in the maize residue retention treatment was significantly higher than in the control. In contrast, the relative abundances of Module #2 (Fig. 3D), #3 (Fig. 3E), and #4 (Fig. 3F) remained unchanged by the maize residue retention. For fungal network, the different modules contained distinct fungal phyla. Ascomycota was the dominant phylum in the Module #1, #2 and #4, and Basidiomycota dominated the Module #3 (Fig. 4B). Maize residue retention significantly enhanced the relative abundance of Module #3 (Fig. 4E), but reduced the abundance of Module #1 (Fig. 4C), #2 (Fig. 4D) and #4 (Fig. 4F) in the fungal network. However, FT intensity exhibited no effect on these modules.

**Figure 5:**
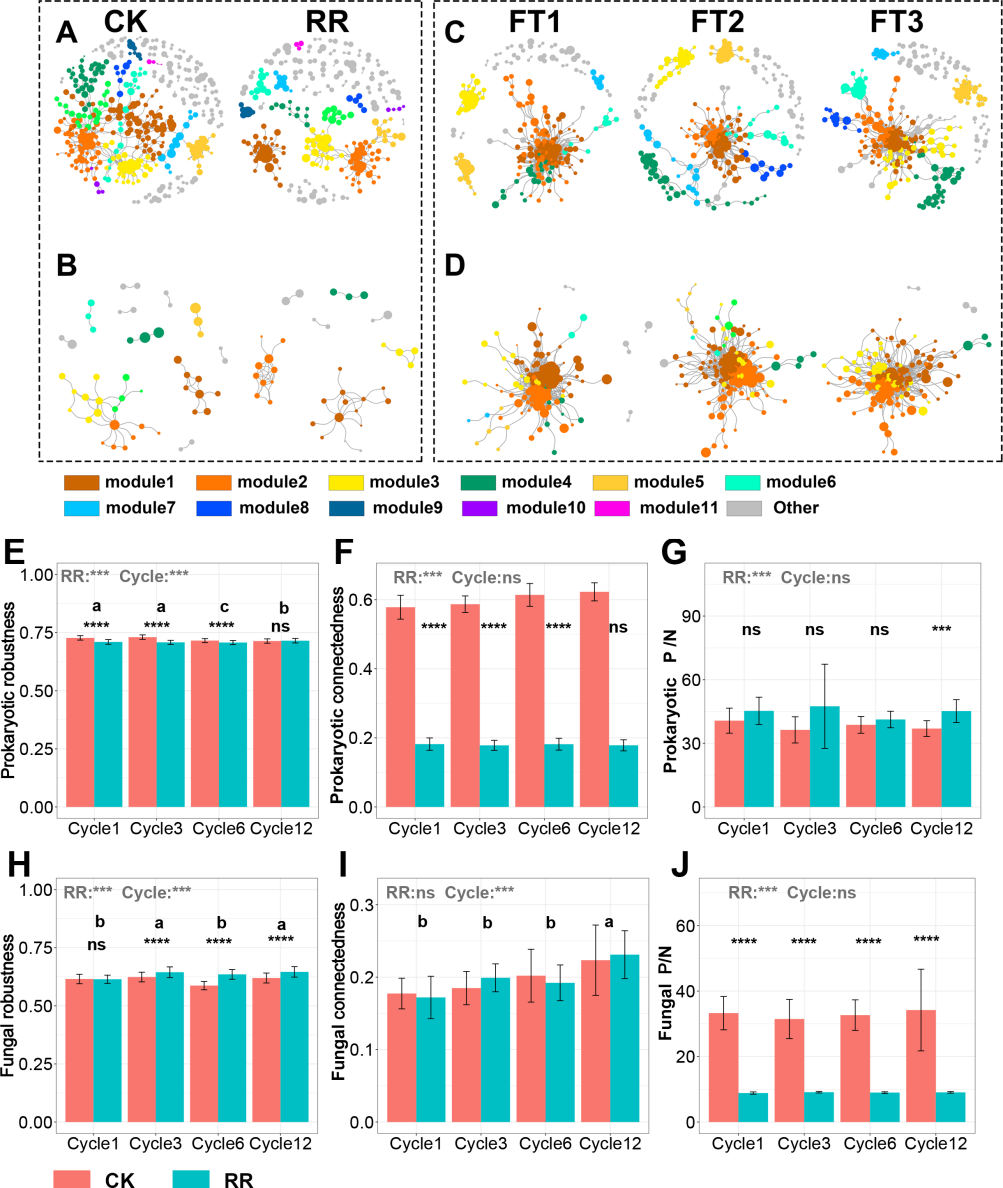
Prokaryotic co-occurrence network in control and maize residue retention (A) treatments; fungal co-occurrence network in control and maize residue retention (B) treatments. Prokaryotic co-occurrence network in FT1, FT2 and FT3 treatments (C); fungal co-occurrence network in FT1, FT2 and FT3 treatments (D). Robustness (E), connectedness (F) and positive links to negative links ratio (P/N ratio, G) of prokaryotic network among treatments; fungal robustness (H), connectedness (I) and positive links to negative links ratio (P/N ratio, J) of fungal network among treatments. In (E–J), symbols indicate the *P* values from t test: ns, not significant; *, 0.01 < *P* < 0.5; **, 0.001 < *P* < 0.01; ***, 0.0001 < *P* < 0.001; ****, *P* < 0.0001. Bars without shared letters indicate significant difference among cycles at *P* < 0.05. Abbreviations: P/N, positive links/negative links ratio; CK, control; RR, maize residue retention; Cycle, freeze-thawing cycles; FT, freeze-thawing; FT1, constant 4 °C; FT2, −4 °C/4 °C (moderate FT intensity), FT3, −10 °C/4 °C (severe FT intensity). Cycle1, Cycle3, Cycle6 and Cycle12 represents for one, three, six and 12 freeze-thawing cycles, respectively.

The visualized prokaryotic networks were smaller and less connected in maize residue retention than in control treatment, while the fungal networks did not display marked difference between treatments (Figs. 5A, 5B, 5C, 5D). These patterns were further supported by the topological properties (*e.g.*, connectedness) calculated based on the whole prokaryotic and fungal networks. Maize residue retention significantly reduced the robustness (Fig. 5E) and connectedness (Fig. 5F) of prokaryotic network, suggesting that the complexity and stability of prokaryotic network decreased after maize residue retention. Although maize residue retention did not affect the connectedness (Fig. 5I) of fungal network, it significantly increased the network robustness (Fig. 5H). We then inferred the interaction relationships among prokaryotes and fungi by calculating the number of positive and negative links in their networks. The positive/negative links (P/N) ratio of prokaryotic network (Fig. 5G) was significantly increased by maize residue retention, but the fungal network exhibited opposite trend (Fig. 5H).

Keystone prokaryotes and fungi were identified based on Pi and Zi value in each treatment. The keystone prokaryotes and fungi were different among control, moderate and severe FT treatments. Notably, fungal network in severe FT treatment harbored more keystone taxa (18) than control (five) and moderate FT treatment (three). The annotation of each keystone taxa is shown in Table S4.

### Function of microbial communities in the soil

**Figure 6:**
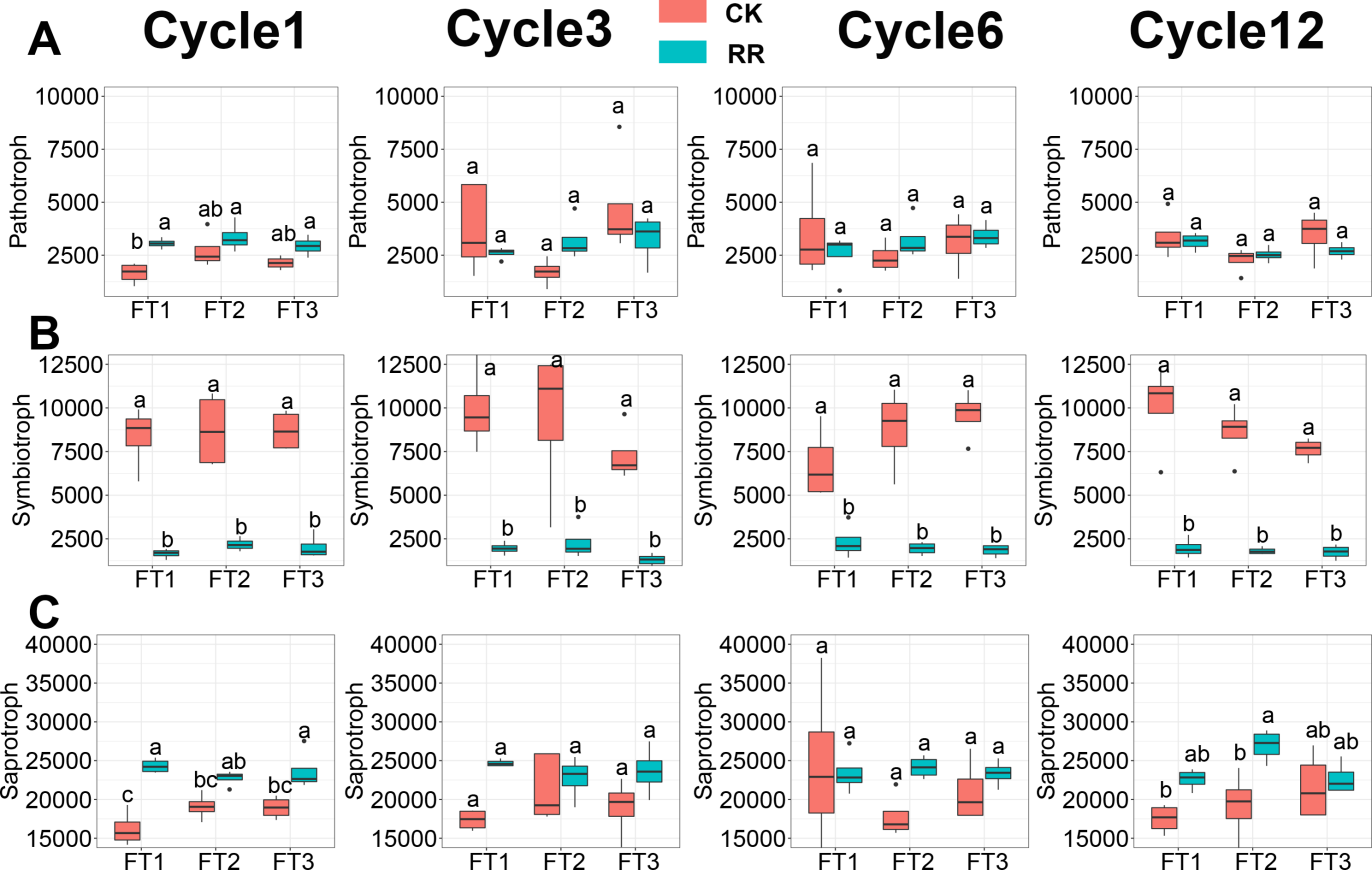
Abundance of fungal pathotroph (A), symbiotroph (B), saprotroph(C) among treatments in Cycle1, Cycle3, Cycle6 and Cycle12. In (A–C), box plots without shared letters indicate significant difference at *P*< 0.05. Abbreviations: CK, control; RR, maize residue retention; Cycle, freeze-thawing cycles; FT, freeze-thawing; FT1, constant 4 °C; FT2, −4 °C/ 4 °C (moderate FT intensity), FT3, −10 °C/ 4 °C (severe FT intensity). Cycle1, Cycle3, Cycle6 and Cycle12 represents for one, three, six and 12 freeze-thawing cycles, respectively.

Soil fungal community was assessed in terms of fungal guilds, and 34.1% of fungal ASVs were assigned to a fungal guild. ANOVA analysis revealed that the abundance of pathotroph (Fig. 6A) and symbiotroph (Fig. 6B) saprotroph (Fig. 6C) were all significantly impacted by maize residue retention and its interaction with the FT cycles. The relative abundance of saprotroph (Fig. 6C) was enhanced by maize residue retention in the 6th FT cycle and 12th FT cycle, but the pathotroph (Fig. 6A) was only enhanced in the 1st FT cycle. Moreover, the relative abundance of symbiotroph (Fig. 6B) were consistently reduced by maize residue retention.

FAPROTAX analysis showed that 39.5% of all bacterial ASVs were assigned to at least one ecological type, and functions related to C and N cycling were the most abundant. The abundance of cellulolysis and nitrification were enhanced by residue retention, but the effect was only statistically significant in 12th cycle (cellulolysis, Fig. S3A) and 3rd cycle (nitrification, Fig. S3B). Although the abundance of denitrification (Fig. S3C) was reduced by residue retention in the first cycle, it was dramatically enhanced by residue retention in the 3rd, 6th and 12th FT cycles.

**Figure 7:**
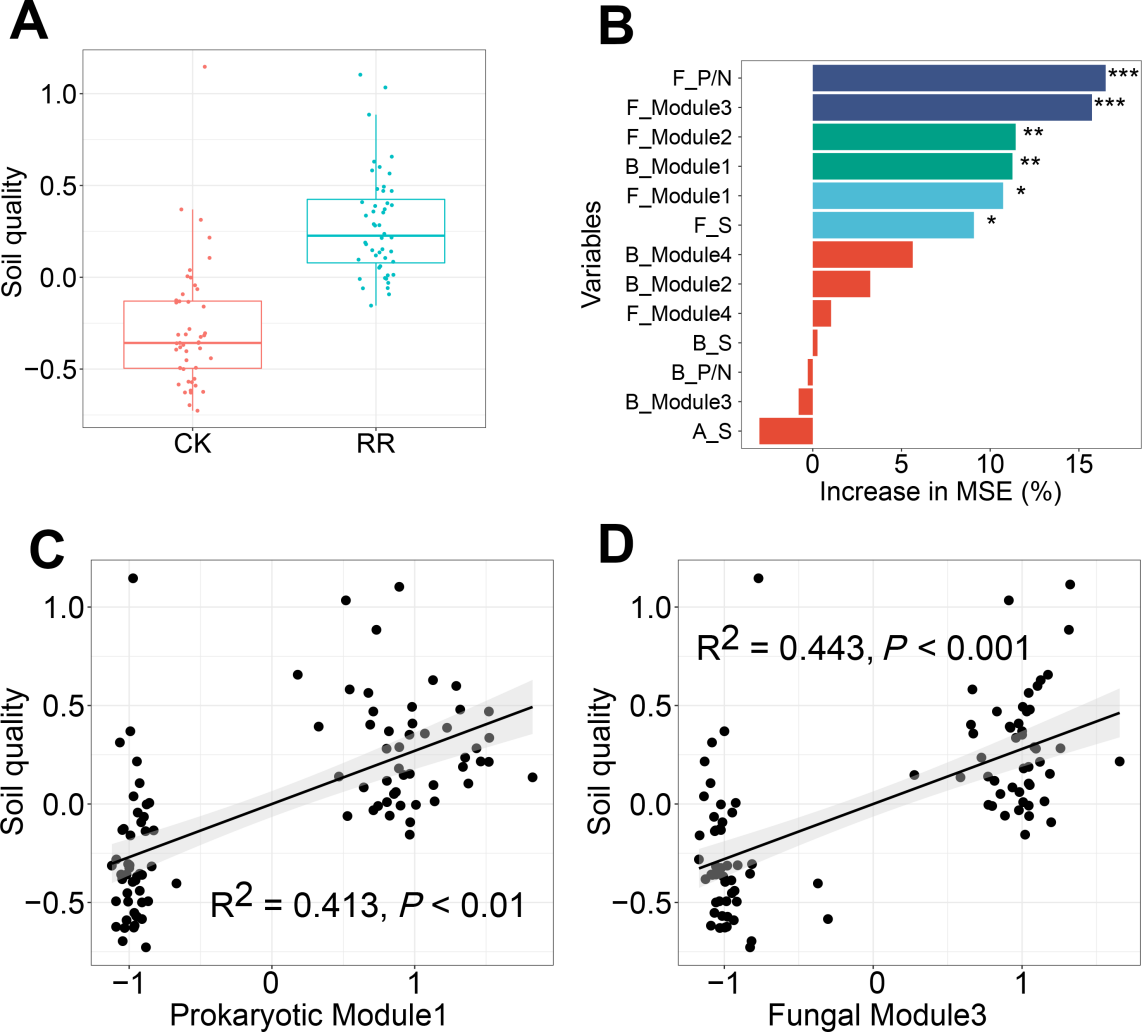
Box plots showing the soil quality in control (CK) and maize residue retention (RR) treatments (A). Random forest mean predictor importance (percentage of increase of mean square error) of archaeal, bacterial and fungal alpha-diversity and network indices as drivers for the soil quality (B), symbols indicate the *P* values: ns, not significant; *, 0.01 <*P* < 0.05; **, 0.001 <*P* < 0.01; ***, 0.0001 <*P* < 0.001. The linear regressions between the relative abundance of soil prokaryotic Module #1 (C) and fungal Module #3 (D) and soil quality. Abbreviations: F, fungal; B, bacterial; A, archaeal; S, richness; P/N, positive links/negative links ratio.

Soil quality was comprehensively assessed using multiple variables including soil nutrient availabilities and enzyme activities. Independent-sample *t* test analysis revealed that soil quality was significantly improved by maize residue retention (Fig. 7A). Random forest model was performed to identify the key factors in predicting soil quality, and explained 40% of the variations in soil quality. Fungal positive links to negative links (P/N) ratio was the most important determinant for soil quality, followed by fungal Module #3, fungal Module #2, prokaryotic Module #1, Module #1 and fungal richness (Fig. 7B). Especially, soil quality had strong and positive correlations with the relative abundance of fungal Module #3 and prokaryotic Module #1 (Figs. 7C, 7D).

## Discussion

### Maize residue retention altered soil microbial communities

Our study is a short-term microcosm study which simulate maize residue retention under different FT intensity across 12 FT cycles. Our results indicated that soil archaeal, bacterial and fungal community compositions were significantly impacted by maize residue retention, and this effect of independent of study sites. This finding was supported by a large number of field studies (Liu et al., 2021; Wang et al., 2018; Xia et al., 2018). For instance, Xu et al. (2023) reported that residue retention induced significant shift in soil microbial communities in three maize fields from north to central China (Xu et al., 2023). Maize straw is rich in labile and recalcitrant organic carbon, thus provide substrates for soil microbes and reshape their community compositions (Wu et al., 2023). Alternatively, maize residue retention would possibly alter the soil microbial communities through the change in soil physiochemical characteristics. As revealed by Mantel test, soil microbial communities were correlated with a series of soil physiochemical variables (*e.g.*, available potassium (AK), available phosphorus (AP), soil total nitrogen (TN)) in the present study.

Maize residue retention induced a shift from oligotrophic-dominated community to copiotrophic-dominated community. The copiotrophic groups including Firmicutes and Bacteroidota, which have high growth rates under resource-rich conditions (Fierer, Bradford & Jackson, 2007), were enriched by maize residue retention (Fig. 1E). It is true that there will be dead cells in the soil after FT treatment, but this will only affect the identification of the presence or absence of species, and will not have much effect on the relative abundance comparison. In contrast, Acidobacteria, Chloroflexi, Verrucomicrobiota, which have oligotrophic attributes, were reduced by maize residue retention (Fig. 1E). The shift of fungal community was also reflected on the functional guilds (Fig. 6). The saprotroph, which involved in the decomposition of complex macromolecules such as cellulose and lignin (Kang et al., 2023), was enriched in maize residue retention treatment (Fig. 6C), and this pattern was increasingly obvious along with the incubation time. This result confirmed the importance of saprotroph in straw decomposition, and implied saprotroph would be increasingly important during the straw decomposition. One concern for farmers in adopting maize residue retention practice is its potential increase in incidence of plant disease (Tang et al., 2011). However, our results indicated that maize residue retention only briefly increased the pathotroph (Fig. 6A) abundance in the first FT cycle, which suggested that maize residue retention practice will not threaten crop health (Govaerts et al., 2006; Wang et al., 2020; Wang et al., 2021).

Contrary to previous studies (Guan et al., 2022; Muhammad et al., 2021), maize residue retention depressed soil archaeal, bacterial and fungal diversity in the current study. The lower soil microbial diversity in maize residue retention treatment can be attributed to the increased importance of deterministic process. Because in communities with large populations, the assembly processes are more susceptible to deterministic process (Xun et al., 2019). Specifically, the decreased richness mainly belonged to the phylum that defined as oligotroph. Therefore, the maize residue retention may act as a selection pressure, and probably caused the decrease in the microbial diversity *via* the disfavour of the oligotrophic groups.

### FT intensity decreased soil microbial activity without affecting their community compositions

FT cycles is a common phenomenon and important factor that leads to soil degradation in black soil region. Our study indicated that moderate and severe FT significantly reduced soil respiration (Fig. S1). FT cycles may depress soil microbial activity directly by lysis of soil microbes or indirectly by disturbance of soil aggregates (Ji et al., 2022; Zong et al., 2023). However, the effect of FT cycles on soil respiration was not detectable under maize residue retention, indicating that maize residue retention would alleviate the adverse effect of FT cycles on soil microbial activity.

Our first hypothesis that soil microbial communities and co-occurrence networks would be affected by maize residue retention and FT was only partially supported by our findings, we found that FT cycles had no significant effect on soil microbial diversity and community composition. This finding was consistent with some studies, which find minor or no detectable effects of FT cycles on soil microbial communities (Männistö, Tiirola & Häggblom, 2009; Meisner et al., 2021). Firstly, although repeated FT would directly reduce soil microbial biomass and diversity, it also release nutrients to soils from dead microbial cells and soil aggregates which would trigger the growth of soil microbes after thawing (Haei et al., 2011; Han et al., 2018). These effects may offset each other. Secondly, soil microbial communities developed in high-altitude or high-latitude regions are reported to be cold-tolerant (Koponen et al., 2006; Yergeau & Kowalchuk, 2008) and resistant to repeated FT (Pastore et al., 2023). Alternatively, the shift of soil microbial communities under FT may be reflected at the gene expression level but not at the DNA replication level (Woodcroft et al., 2018). Because soil microorganisms can enter a dormant state under FT, and their 16s rDNA or ITS fragments can still be detected by amplicon sequencing (Woodcroft et al., 2018).

### Soil microbial network was affected by maize residue retention rather than FT intensities

Since organic inputs provide a substantial supply of substrates and nutrients for soil microbes, previous studies indicated that organic inputs generally increased the complexity of soil microbial networks (Yang et al., 2019). However, we observed that maize residue retention simplified soil prokaryotic network, reflected by the greater number of nodes, links and connectedness. The simplified prokaryotic network is not likely due to the increased nutrient availabilities, but more likely to be the consequence of disturbed microhabitats and fragmented niches after maize straw incorporated in soils. Simple networks with smaller connectivity are generally less resistant to environmental perturbations than complex networks (Xu et al., 2023). Herein, the robustness of prokaryotic network (Fig. 5E) was also reduced by maize residue retention. Fungal network (Fig. 5H) exhibited different pattern as compared with prokaryotic network. Although maize residue retention did not affect fungal network complexity (Fig. 5I), it dramatically enhanced the network stability (Fig. 5H). This result also collaborated with the finding that maize residue retention decreased fungal P/N ratio (Fig. 5J). As proposed by Coyte, Schluter & Foster (2015), the negative interactions among members might stabilize co-oscillation in communities and promote stability of networks (Coyte, Schluter & Foster, 2015). Taken as a whole, our findings suggested that soil fungal community would be resistant against environmental stresses under maize residue retention.

Although FT intensity did not impact the network pattern of soil prokaryoates (Fig. 5C) and fungi (Fig. 5D), it altered the keystone taxa in network (Table S4). The keystone prokaryotes and fungi were totally different among control, moderate and severe FT treatments. Especially, the fungal network in severe FT treatment harbored the most abundant keystone taxa. Among these keystone taxa, *Pseudogymnoascus roseus* and *Pseudeurotium hygrophilum* were reported to be cold-adapted fungi (Ramasamy et al., 2023), and thereby may stabilize fungal network under repeated severe FT.

### Potential roles of network modules in driving soil quality

The effect of maize residue retention on soil function is still a subject of considerable debate (Wu et al., 2023). Our results proved that maize residue retention would improve soil quality (Fig. 7). Especially, the enzyme that related to straw decomposition including β-D-glucosidase (BG) and β-D-xylosidase (XYL), were significantly enhanced by residue retention (Table S1). The residue retention would trigger the growth of soil microbes, thus providing a favorable environment for the accumulation of soil enzyme. We then explored the key factors that contribute to soil quality. Recently, a large number of research have recorded that soil quality is positively correlated with soil microbial diversity (Delgado-Baquerizo et al., 2016; Mori et al., 2016; Wagg et al., 2019). The current study proposed that network modules were more important than microbial diversity in predicting soil quality, suppporting our third hypothesis. Modules identified in the network represent a group of microbial taxa that potentially interact or share similar niches (Wiens, 2010), and contribute to specific ecological processes (Xu et al., 2023). We found that the main modules exhibit different strategies to maize residue retention. Module #1 in prokaryotic network (Fig. 3B), which consists of multiple phylum, positively responded to maize residue retention. Members in this module are capable of celloluse degradation (*e.g.*, *Bacillus* and *Cellulosimicrobium*), lignin degradation (*e.g.*, *Streptomyces* and *Paenibacillus*), participate in N cycling (*e.g.*, *Burkholderia*). These members interacted with each other and would be efficient in straw degradation. Likewise, Module #3 in fungal network (Fig. 4B) also exhibited a great preference to maize residue retention. Interestingly, more than half members in fungal Module #3 belonged to genus *Chaetomium*, *Rhizopus* and *Mucor*, which are typical cellulose-degrading fungi (Ferreira et al., 2013; Liu et al., 2021). These results indicated that members in these two modules would involve in processes that related to maize residue retention. However, the relative abundance of fungal Module #1, 2, and 4 were sharply decreased by maize residue retention. These modules possibly either be depressed by the unfavorable condition (*e.g.*, anaerobic condition) created by maize residue retention or due to the aggravated competition (Guan et al., 2022).

## Conclusions

In conclusion, our results indicated that maize residue retention induced pronounced changes in soil microbial communities and significantly reduced their richness. FT cycles also impacted soil physiochemical properties, enzyme activities and the soil microbial community. Although FT intensity did not impact soil microbial diversity and community composition, it depressed soil respiration without maize residue retention. Moreover, maize residue retention reduced the complexity and stability of soil prokaryotic network, while improved fungal network stability, indicating a high resistance of fungal communities to maize residue retention. Taken as a whole, our results indicated that maize residue retention is a stronger determinant than FT intensity in shaping soil microbial communities in black soil region, and highlighted that the network modules contributed more to soil quality than microbial diversity.

## Supplemental Information

10.7717/peerj.17543/supp-1 Supplemental Information 1 Supplementary Figures and Tables

10.7717/peerj.17543/supp-2 Supplemental Information 2 Code of diversity analysis and network analysis

10.7717/peerj.17543/supp-3 Supplemental Information 3 Raw data

10.7717/peerj.17543/supp-4 Supplemental Information 4 Author change justification

## Additional Information and Declarations

### DNA Deposition

The following information was supplied regarding the deposition of DNA sequences: The raw sequences are available at NCBI SRA: PRJNA1045363, SRR26950863 –SRR26951054).

### Data Availability

The following information was supplied regarding data availability: The raw data is available to the Supplementary File.

## References

[ref-1] Amir A, McDonald D, Navas-Molina JA, Kopylova E, Morton JT, Xu ZZ, Kightley EP, Thompson LR, Hyde ER, Gonzalez A, Knight R (2017). Deblur rapidly resolves single-nucleotide community sequence patterns. mSystems.

[ref-2] Bao SD (2000). Soil and agricultural chemistry analysis.

[ref-3] Brown JH, Gillooly JF, Allen AP, Savage VM, West GB (2004). Toward a metabolic theory of ecology. Ecology.

[ref-4] Caporaso JG, Kuczynski J, Stombaugh J, Bittinger K, Bushman FD, Costello EK, Fierer N, Peña AG, Goodrich JK, Gordon JI, Huttley GA, Kelley ST, Knights D, Koenig JE, Ley RE, Lozupone CA, McDonald D, Muegge BD, Pirrung M, Reeder J, Sevinsky JR, Tumbaugh PJ, Walters WA, Widmann J, Yatsunenko T, Zaneveld J, Knight R (2010). QIIME allows analysis of high-throughput community sequencing data. Nature Methods.

[ref-5] Chen H, Chen Z, Chu XY, Deng Y, Qing SQ, Sun CR, Wang Q, Zhou HB, Cheng HN, Zhan WH, Wang YG (2022). Temperature mediated the balance between stochastic and deterministic processes and reoccurrence of microbial community during treating aniline wastewater. Water Research.

[ref-6] Chen J, Li Z, Xu D, Xiao Q, Liu H, Li X, Chao L, Qu H, Zheng Y, Liu X, Wang P, Bao Y (2023). Patterns and drivers of microbiome in different rock surface soil under the volcanic extreme environment. iMeta.

[ref-7] Coyte KZ, Schluter J, Foster KR (2015). The ecology of the microbiome: networks, competition, and stability. Science.

[ref-8] Csardi G, Nepusz T (2006). The igraph software package for complex network research. InterJournal.

[ref-9] De Forest JL (2009). The influence of time, storage temperature, and substrate age on potential soil enzyme activity in acidic forest soils using MUB-linked substrates and L-DOPA. Soil Biology & Biochemistry.

[ref-10] de Vries FT, Griffiths RI, Bailey M, Craig H, Girlanda M, Gweon HS, Hallin S, Kaisermann A, Keith AM, Kretzschmar M, Lemanceau P, Lumini E, Mason KE, Oliver A, Ostle N, Prosser JI, Thion C, Thomson B, Bardgett RD (2018). Soil bacterial networks are less stable under drought than fungal networks. Nature Communications.

[ref-11] Delgado-Baquerizo M, Maestre FT, Reich PB, Jeffries TC, Gaitan JJ, Encinar D, Berdugo M, Campbell CD, Singh BK (2016). Microbial diversity drives multifunctionality in terrestrial ecosystems. Nature Communications.

[ref-12] Deng Y, Jiang YH, Yang Y, He Z, Luo F, Zhou J (2012). Molecular ecological network analyses. BMC Bioinformatics.

[ref-13] Fernley, Walker (1965). Kinetic behaviour of calf-intestinal alkaline phosphatase with 4-methylumbelliferyl phosphate.

[ref-14] Ferreira JA, Lennartsson PR, Edebo L, Taherzadeh MJ (2013). Zygomycetes-based biorefinery: present status and future prospects. Bioresource Technology.

[ref-15] Fierer N (2017). Embracing the unknown: disentangling the complexities of the soil microbiome. Nature Reviews Microbiology.

[ref-16] Fierer N, Bradford MA, Jackson RB (2007). Toward an ecological classification of soil bacteria. Ecology.

[ref-17] Fujita T, Noguchi K, Terashima I (2013). Apoplastic mesophyll signals induce rapid stomatal responses to CO2 in Commelina communis. New Phytologist.

[ref-18] Goslee SC, Urban DL (2020). The ecodist package for dissimilarity-based analysis of ecological data. Journal of Statistical Software.

[ref-19] Govaerts B, Mezzalama M, Sayre KD, Crossa J, Nicol JM, Deckers J (2006). Long-term consequences of tillage, residue management, and crop rotation on maize/wheat root rot and nematode populations in subtropical highlands. Applied Soil Ecology.

[ref-20] Groffman PM, Hardy JP, Fashu-Kanu S, Driscoll CT, Cleavitt NL, Fahey TJ, Fisk MC (2010). Snow depth, soil freezing and nitrogen cycling in a northern hardwood forest landscape. Biogeochemistry.

[ref-21] Gu SY, Guo XJ, Cai YT, Zhang ZH, Wu S, Li X, Zhang HH, Yang W (2018). Residue management alters microbial diversity and activity without affecting their community composition in black soil, Northeast China. PeerJ.

[ref-22] Gu SY, Wu S, Guan YP, Zhai C, Zhang ZH, Bello A, Guo XJ, Yang W (2020). Arbuscular mycorrhizal fungal community was affected by tillage practices rather than residue management in black soil of northeast China. Soil & Tillage Research.

[ref-23] Guan Y, Xu B, Zhang X, Yang W (2022). Tillage practices and residue management manipulate soil bacterial and fungal communities and networks in maize agroecosystems. Microorganisms.

[ref-24] Guimerà R, Amaral LAN (2005). Cartography of complex networks:: modules and universal roles. Journal of Statistical Mechanics-Theory and Experiment.

[ref-25] Haei M, Rousk J, Ilstedt U, Öquist M, Bååth E, Laudon H (2011). Effects of soil frost on growth, composition and respiration of the soil microbial decomposer community. Soil Biology & Biochemistry.

[ref-26] Han ZM, Deng MW, Yuan AQ, Wang JH, Li H, Ma JC (2018). Vertical variation of a black soil’s properties in response to freeze-thaw cycles and its links to shift of microbial community structure. Science of The Total Environment.

[ref-27] Ihrmark K, Bödeker ITM, Cruz-Martinez K, Friberg H, Kubartova A, Schenck J, Strid Y, Stenlid J, Brandström-Durling M, Clemmensen KE, Lindahl BD (2012). New primers to amplify the fungal ITS2 region - evaluation by 454-sequencing of artificial and natural communities. FEMS Microbiology Ecology.

[ref-28] Ji X, Liu M, Yang J, Feng F (2022). Meta-analysis of the impact of freeze–thaw cycles on soil microbial diversity and C and N dynamics. Soil Biology and Biochemistry.

[ref-29] Ji YC, Wang DY (2022). Durability of recycled aggregate concrete in cold regions. Case Studies in Construction Materials.

[ref-30] Jordan F (2009). Keystone species and food webs. Philosophical Transactions of the Royal Society B.

[ref-31] Kang P, Pan YQ, Ran YC, Li WA, Shao MX, Zhang YQ, Ji QB, Ding XD (2023). Soil saprophytic fungi could be used as an important ecological indicator for land management in desert steppe. Ecological Indicators.

[ref-32] Koponen HT, Jaakkola T, Keinänen-Toivola MM, Kaipainen S, Tuomainen J, Servomaa K, Martikainen PJ (2006). Microbial communities, biomass, and activities in soils as affected by freeze thaw cycles. Soil Biology & Biochemistry.

[ref-33] Liu JG, Diamond J (2005). China’s environment in a globalizing world. Nature.

[ref-34] Liu L, Huang W-C, Liu Y, Li M (2021). Diversity of cellulolytic microorganisms and microbial cellulases. International Biodeterioration & Biodegradation.

[ref-35] Liu JJ, Zhang KD, Shi WB, Liu LJ, Lu C (2024). Effects of freeze-thaw on soil detachment capacity in the black soil region of Northeastern China. Soil & Tillage Research.

[ref-36] Männistö MK, Tiirola M, Häggblom MM (2009). Effect of freeze-thaw cycles on bacterial communities of Arctic Tundra soil. Microbial Ecology.

[ref-37] Meisner A, Snoek BL, Nesme J, Dent E, Jacquiod E, Classen AT, Priemé A (2021). Soil microbial legacies differ following drying-rewetting and freezing-thawing cycles. ISME Journal.

[ref-38] Mori AS, Isbell F, Fujii S, Makoto K, Matsuoka S, Osono T (2016). Low multifunctional redundancy of soil fungal diversity at multiple scales. Ecology Letters.

[ref-39] Muhammad I, Wang J, Sainju UM, Zhang SH, Zhao FZ, Khan A (2021). Cover cropping enhances soil microbial biomass and affects microbial community structure: a -analysis. Geoderma.

[ref-40] Nguyen NH, Song ZW, Bates ST, Branco S, Tedersoo L, Menke J, Schilling JS, Kennedy PG (2016). FUNGuild: an open annotation tool for parsing fungal community datasets by ecological guild. Fungal Ecology.

[ref-41] Ning DL, Deng Y, Tiedje JM, Zhou JZ (2019). A general framework for quantitatively assessing ecological stochasticity. Proceedings of the National Academy of Sciences of the United States of America.

[ref-42] Oksanen J, Blanchet FG, Friendly M, Kindt R, Legendre P, McGlinn D, Minchin PR, O’Hara RB, Simpson GL, Solymos P, Stevens MHH, Szoecs E, Wagner H (2020). vegan: Community ecology package. https://CRAN.R-project.org/package=vegan.

[ref-43] Ouyang W, Shan YS, Hao FH, Chen SY, Pu X, Wang MK (2013). The effect on soil nutrients resulting from land use transformations in a freeze-thaw agricultural ecosystem. Soil & Tillage Research.

[ref-44] Pastore MA, Classen AT, English ME, Frey SD, Knorr MA, Rand K, Adair EC (2023). Soil microbial legacies influence freeze–thaw responses of soil. Functional Ecology.

[ref-45] Pinheiro J, Bates D, DebRoy S, Sarkar D (2013). https://CRAN.R-project.org/package=nlme.

[ref-46] R Core Project (2023). R: a language and environment for statistical computing. https://www.R-project.org.

[ref-47] Ramasamy KP, Mahawar L, Rajasabapathy R, Rajeshwari K, Miceli C, Pucciarelli S (2023). Comprehensive insights on environmental adaptation strategies in Antarctic bacteria and biotechnological applications of cold adapted molecules. Frontiers in Microbiology.

[ref-48] Saiya-Cork KR, Sinsabaugh RL, Zak DR (2002). The effects of long term nitrogen deposition on extracellular enzyme activity in an forest soil. Soil Biology & Biochemistry.

[ref-49] Shen QH, Suarez-Abelenda M, Camps-Arbestain M, Pereira RC, McNally SR, Kelliher F (2018). Data on the organic matter characteristics of New Zealand soils under different land uses. Data in Brief.

[ref-50] Soil Survey Staff (2022). Keys to Soil Taxonomy, 13th edition.

[ref-51] Tang H-M, Xiao X-P, Tang W-G, Yang G-L (2011). Effects of straw recycling of winter covering crop on methane and nitrous oxide emissions in paddy field. Acta Agronomica Sinica.

[ref-52] Wagg C, Schlaeppi K, Banerjee S, Kuramae EE, van der Heijden MGA (2019). Fungal-bacterial diversity and microbiome complexity predict ecosystem functioning. Nature Communications.

[ref-53] Wang QJ, Cao X, Jiang H, Guo ZH (2021). Straw application and soil microbial biomass carbon change: a meta-analysis. Clean-Soil Air Water.

[ref-54] Wang H, Li X, Li X, Wang J, Li X, Guo Q, Yu Z, Yang T, Zhang H (2020). Long-term no-tillage and different residue amounts alter soil microbial community composition and increase the risk of maize root rot in northeast China. Soil and Tillage Research.

[ref-55] Wang M, Pendall E, Fang CM, Li B, Nie M (2018). A global perspective on agroecosystem nitrogen cycles after returning crop residue. Agriculture Ecosystems & Environment.

[ref-56] Wasimuddin K, Schlaeppi, Ronchi F, Leib SL, Erb M, Ramette A (2020). Evaluation of primer pairs for microbiome profiling from soils to humans within the One Health framework. Molecular Ecology Resources.

[ref-57] Wei WJ, You WZ, Zhang HD, Yan TW, Mao YX (2016). Soil respiration during freeze-thaw cycles in a temperate Korean Larch (*Larix olgensis* herry.) plantation. Scandinavian Journal of Forest Research.

[ref-58] Wiens DP (2010). Robustness of design for the testing of lack of fit and for estimation in binary response models. Computational Statistics & Data Analysis.

[ref-59] Woodcroft BJ, Singleton CM, Boyd JA, Evans PN, Emerson JB, Zayed AAF, Hoelzle RD, Lamberton TO, McCalley CK, Hodgkins SB, Wilson RM, Purvine SO, Nicora CD, Li C, Frolking S, Chanton JP, Crill PM, Saleska SR, Rich VI, Tyson GW (2018). Genome-centric view of carbon processing in thawing permafrost. Nature.

[ref-60] Wu G, Ling J, Zhao D-Q, Liu Z-X, Xu Y-P, Kuzyakov Y, Marsden K, Wen Y, Zhou S-L (2023). Straw return counteracts the negative effects of warming on microbial community and soil multifunctionality. Agriculture, Ecosystems & Environment.

[ref-61] Xia LL, Lam SK, Wolf B, Kiese R, Chen DL, Butterbach-Bahl K (2018). Trade-offs between soil carbon sequestration and reactive nitrogen losses under straw return in global agroecosystems. Global Change Biology.

[ref-62] Xu Z, Sun R, He T, Sun Y, Wu M, Xue Y, Meng F, Wang J (2023). Disentangling the impact of straw incorporation on soil microbial communities: enhanced network complexity and ecological stochasticity. Science of The Total Environment.

[ref-63] Xun WB, Li W, Xiong W, Ren Y, Liu YP, Miao YZ, Xu ZH, Zhang N, Shen QR, Zhang RF (2019). Diversity-triggered deterministic bacterial assembly constrains community functions. Nature Communications.

[ref-64] Yanai Y, Toyota K, Okazaki M (2011). Effects of successive soil freeze-thaw cycles on nitrification potential of soils. Soil Science and Plant Nutrition.

[ref-65] Yang W, Jing XY, Guan YP, Zhai C, Wang T, Shi DY, Sun WP, Gu SY (2019). Response of fungal communities and co-occurrence network patterns to compost amendment in black soil of Northeast China. Frontiers in Microbiology.

[ref-66] Yang XC, Zhu K, Loik ME, Sun W (2021). Differential responses of soil bacteria and fungi to altered precipitation in a meadow steppe. Geoderma.

[ref-67] Yao Q, Liu JJ, Yu ZH, Li YS, Jin J, Liu XB, Wang GH (2017). Changes of bacterial community compositions after three years of biochar application in a black soil of northeast China. Applied Soil Ecology.

[ref-68] Yergeau E, Kowalchuk GA (2008). Responses of Antarctic soil microbial communities and associated functions to temperature and freeze-thaw cycle frequency. Environmental Microbiology.

[ref-69] Yuan MM, Guo X, Wu LW, Zhang Y, Xiao NJ, Ning DL, Shi Z, Zhou XS, Wu LY, Yang YF, Tiedje JM, Zhou JZ (2021). Climate warming enhances microbial network complexity and stability. Nature Climate Change.

[ref-70] Zhang L, Zu C, Yu J, Yan W, Sun N, Tan G, Zhao H, Li F, Meng X, Bian S (2021). Effects of straw returning on soil moisture, temperature and maize yielf in semi humid area. Journal of Soil and Water Conservation.

[ref-71] Zong R, Wang Z, Li W, Ayantobo OO, Li H, Song L (2023). Assessing the impact of seasonal freezing and thawing on the soil microbial quality in arid northwest China. Science of The Total Environment.

