# Supplementary material for: Maize residue retention shapes soil microbial communities and co-occurrence networks upon freeze-thawing cycles
Source: PeerJ. 2024 Jun 14;12:e17543. doi: 10.7717/peerj.17543 (PMC11182024; doi:10.7717/peerj.17543)
Supplement: Supplemental Information 1 [file peerj-12-17543-s001.docx]

**Maize residue retention shapes soil microbial communities and co-occurrence networks upon freeze-thawing cycles**

Yang Yu^1#^, Quankuan Guo^1#^, Shuhan Zhang^2^, Yupeng Guan^2^, Nana Jiang^1^, Yang Zhang^3^, Rong Mao^3^, Keyu Bai^4^, Salimjan Buriyev^5^, Nuriddin Samatov^5^, Ximei Zhang^2*^, Wei Yang^2*^

1 College of Resources and Environment, Northeast Agricultural University, Harbin, 150006, China

2 Institute of Environment and Sustainable Development in Agriculture, Chinese Academy of Agricultural Sciences, Beijing, 100081, China

3 College of Forestry, Jiangxi Agricultural University, Nanchang, 330045, China

4 Alliance of Biodiversity International and CIAT, Beijing office, Institute of Agricultural Resources and Regional Planning, Chinese Academy of Agricultural Sciences, Beijing, 100081, China.

5 Research Institute of Environment and Nature Conservation Technologies of the Ministry of Ecology, Environmental Protection, and Climate Change of the Republic of Uzbekistan, Tashkent, 100043, Uzbekistan.

* Correspondence: zhangximei@caas.cn (X.Z.); yangwei@caas.cn (W.Y.)

# These authors contributed equally to this work.

**
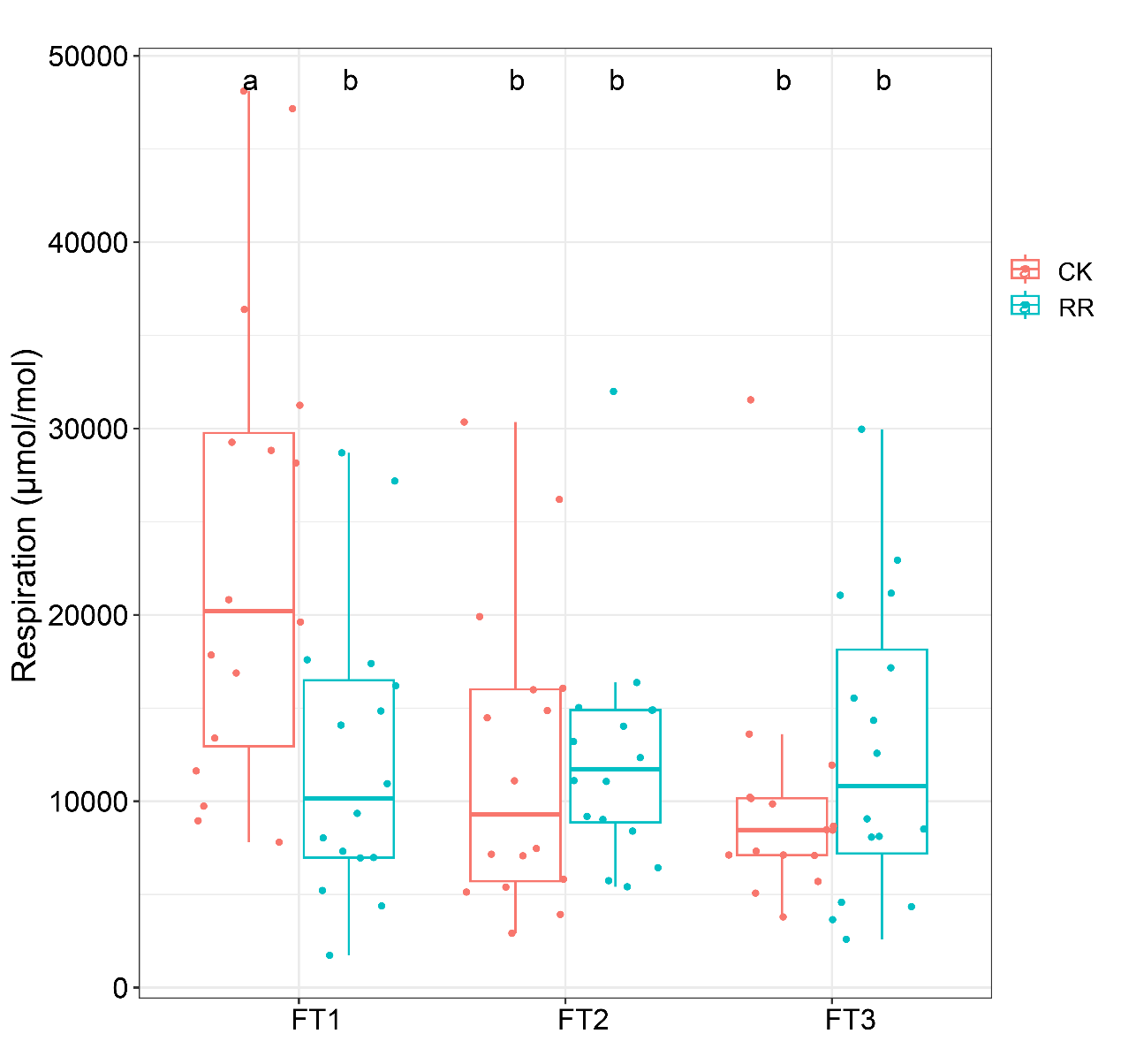
Fig. S1 Soil respiration among treatments.** Box plots without shared letters indicate significant difference at *P* < 0.05. Abbreviations: CK, control; RR, maize residue retention; FT, freeze-thawing; FT1, constant 4 °C; FT2, -4℃/ 4℃ (moderate FT intensity), FT3, -10℃/ 4℃ (severe FT intensity).

**
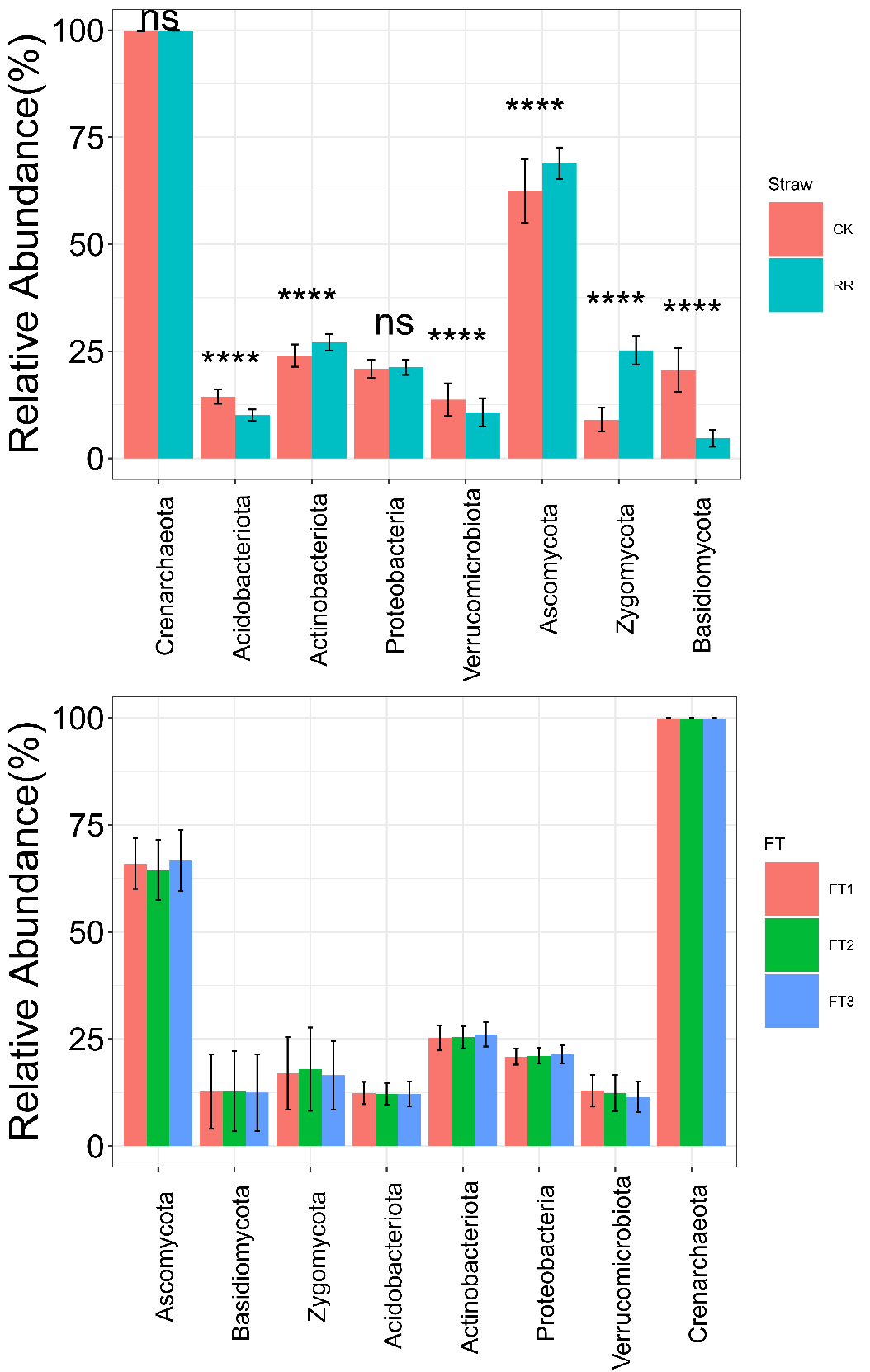
**

Fig. S2 Relative abundance of the main phylum of archaea, bacteria and fungi in CK and RR treatments (A); relative abundance of the main phylum of archaea, bacteria and fungi in FT1, FT2 and FT3 treatments (B). Symbols indicate the *P* values from t test: ns, not significant; *, 0.01 < *P* < 0.5; **, 0.001 < *P* < 0.01; ***, 0.0001 < *P* < 0.001; ****, *P* < 0.0001. Abbreviations: CK, control; RR, maize residue retention; FT, freeze-thawing; FT1, constant 4 °C; FT2, -4℃/ 4℃ (moderate FT intensity), FT3, -10℃/ 4℃ (severe FT intensity).


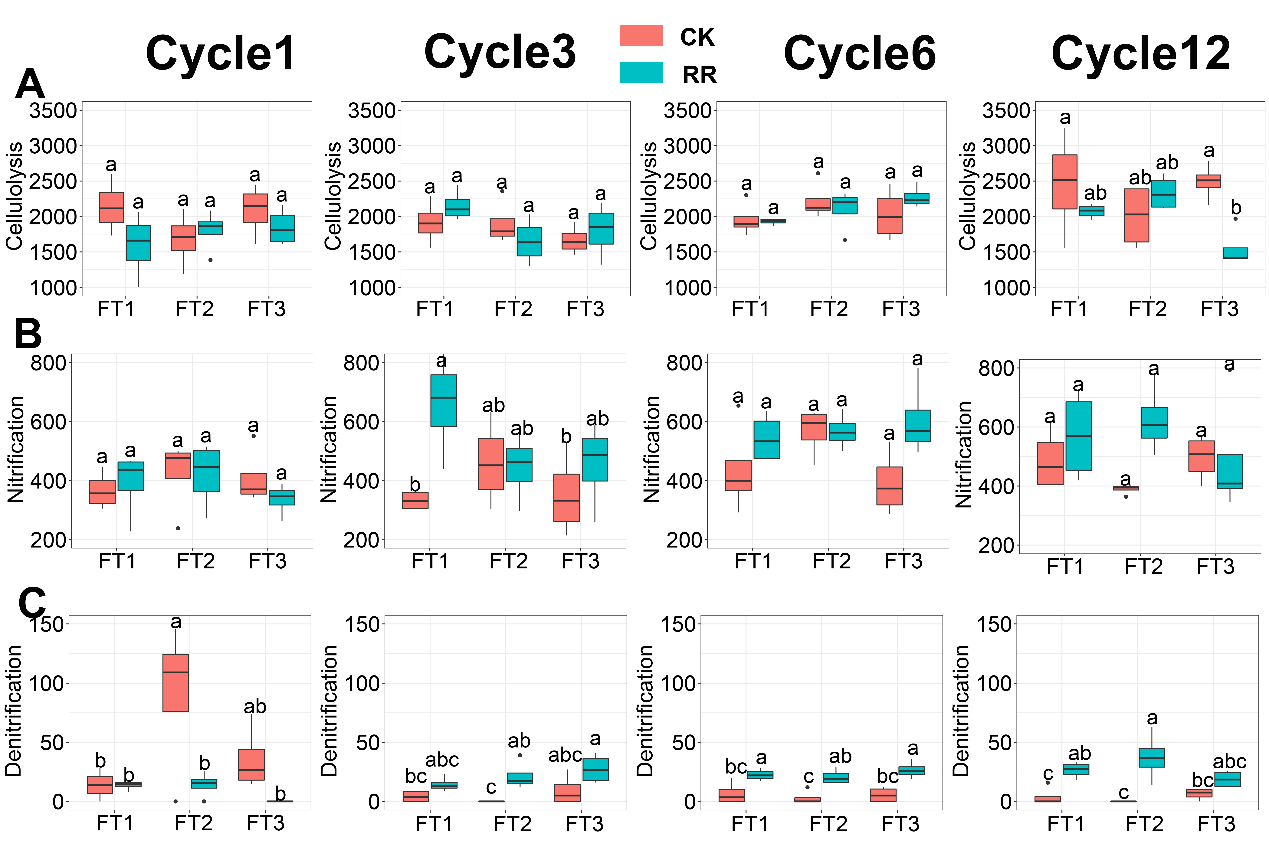
Fig. S3 Abundance of bacterial cellulolysis (A), nitrification (B) and denitrification (C) predicted from FAPROTAX among treatments in Cycle1, Cycle3, Cycle6 and Cycle12. In A-C, box plots without shared letters indicate significant difference at *P* < 0.05. Abbreviations: CK, control; RR, maize residue retention; FT, freeze-thawing; FT1, constant 4 °C; FT2, -4℃/ 4℃ (moderate FT intensity); FT3, -10℃/ 4℃ (severe FT intensity). Cycle1, Cycle3, Cycle6 and Cycle12 represents for one, three, six and 12 freeze-thawing cycles, respectively.

| Table S1 Soil properties, enzyme activity, respiration and richness of bacteria, archaea and fungi among treatments. Values are mean ± SD. Abbreviations: CK, control; RR, maize residue retention; FT, freeze-thawing; FT1, constant 4 °C; FT2, -4℃/ 4℃ (moderate FT intensity); FT3, -10℃/ 4℃ (severe FT intensity). Cycle1, Cycle3, Cycle6 and Cycle12 represents for one, three, six and 12 freeze-thawing cycles, respectively. | | | | | | | | | | | | | | | | | | | |
| --- | --- | --- | --- | --- | --- | --- | --- | --- | --- | --- | --- | --- | --- | --- | --- | --- | --- | --- | --- |
| Residue retention | FT intensity | FT cycles | APE | BG | XYL | LAP | PPO | UE | AK | AP | pH | respiration | NH_4_^+^-N | NO_3_^-^-N | bac_S | arch_S | fun_S | TN | TC |
| CK | 1 | 1 | 28.37±7.55 | 39.17±8.43 | 4.34±1.48 | 4.64±2.35 | 5.92±1.22 | 456.44±25.61 | 335.88±39.23 | 176.28±9.14 | 8.52±0.19 | 40234.06±9040.82 | 31.04±5.76 | 69.46±13.57 | 2313.25±115.29 | 43±6.68 | 897.75±115.23 | 0.14±0 | 1.59±0.06 |
| RR | 1 | 1 | 43.41±18.98 | 56.27±11.51 | 7.39±2.49 | 8.1±1.58 | 3.87±0.39 | 519.3±45.94 | 553.33±21.46 | 206.52±3.86 | 8.22±0.49 | 11024.76±10845.35 | 43.17±4.45 | 59.73±46.37 | 1714±440.66 | 33.5±4.65 | 468.25±83.19 | 0.15±0 | 1.59±0.03 |
| CK | 2 | 1 | 35.74±7.1 | 41.15±6.16 | 8.49±4.79 | 4.5±1.12 | 5.36±2.43 | 455.76±51.49 | 329.81±16.91 | 171.95±3.19 | 7.97±0.12 | 12498.34±7334.76 | 35.86±8.19 | 81.61±39.07 | 2269.25±199.47 | 34.75±3.77 | 854.5±95 | 0.14±0 | 1.54±0.04 |
| RR | 2 | 1 | 39.96±8.43 | 54.75±2.56 | 6.4±1.37 | 6.9±1.15 | 5.31±1.69 | 548.11±18.97 | 592.31±49.15 | 214.13±4.95 | 7.26±0.04 | 8444.48±3367.86 | 27.24±0.83 | 63.63±13.62 | 1958.25±272.59 | 37.25±4.65 | 485.5±33.31 | 0.15±0 | 1.53±0.02 |
| CK | 3 | 1 | 34.59±4.23 | 48.13±5.62 | 6.52±0.21 | 5.28±1.74 | 4.76±1.25 | 467.84±25.28 | 360.94±30.12 | 176.93±5.58 | 7.29±0.07 | 7961.16±2735.29 | 30.49±8.4 | 78.58±43.87 | 2176.25±90.01 | 38±7.44 | 825.75±208.31 | 0.15±0 | 1.67±0.04 |
| RR | 3 | 1 | 45.71±10.89 | 61.77±11.28 | 7.54±2.1 | 6.13±1.45 | 4.96±0.94 | 514.33±39.04 | 536.02±26.91 | 203.52±6.67 | 7.08±0.09 | 23781.9±4212.33 | 40.59±6.76 | 43.66±17.32 | 1945±97.13 | 37.75±2.06 | 505.25±54.71 | 0.15±0 | 1.68±0.09 |
| CK | 1 | 3 | 45.87±9.34 | 44.32±8.26 | 6.66±0.75 | 4.59±1.65 | 6.26±1.55 | 519.3±45.94 | 326.02±11.74 | 163±18.61 | 7.08±0.08 | 23572.08±8122.05 | 52.27±7.8 | 111.01±45.93 | 2428±173.75 | 42.25±3.4 | 778.5±123.02 | 0.14±0 | 1.58±0.02 |
| RR | 1 | 3 | 72.19±24.34 | 59.24±14.52 | 7.74±3.13 | 11.03±11.36 | 4.51±0.63 | 392.22±23.23 | 583.01±47.1 | 207.52±2.85 | 6.61±0.2 | 11843.25±4389.17 | 49.1±16.03 | 88.18±47.95 | 2438.25±113.14 | 36.25±2.99 | 478.25±18.93 | 0.15±0 | 1.58±0.07 |
| CK | 2 | 3 | 51.37±7.09 | 42.85±8.64 | 5.48±0.79 | 3.74±1.02 | 3.73±1.26 | 548.11±18.97 | 328.77±10.23 | 173.96±7.7 | 6.78±0.06 | 10957.17±5208.93 | 39.57±10.22 | 74.36±42.24 | 2456.25±60.92 | 40.75±6.18 | 779.25±124.3 | 0.14±0 | 1.58±0.01 |
| RR | 2 | 3 | 59.73±5.37 | 57.73±3.99 | 7±1.4 | 6.78±0.82 | 4.48±0.36 | 416.1±48.75 | 591.74±14.61 | 203.97±12.62 | 6.24±0.03 | 11884.88±2391.89 | 38.89±10.48 | 77.1±43.01 | 1991.25±313.83 | 29.25±3.77 | 423.5±20.76 | 0.15±0 | 1.54±0.02 |
| CK | 3 | 3 | 51.38±3.91 | 42.48±2.64 | 5.16±0.67 | 3.69±0.31 | 4.47±0.39 | 467.84±25.28 | 387.61±15.49 | 175.29±7.96 | 6.55±0.05 | 8547.77±1241.06 | 43.02±11.61 | 89.28±25.01 | 2137.5±179.94 | 33±4.69 | 752.75±110.68 | 0.14±0 | 1.6±0.03 |
| RR | 3 | 3 | 70.87±7.34 | 78.26±24.1 | 6.99±1.1 | 10.39±6.05 | 3.81±0.61 | 390.42±28.67 | 699.41±69.26 | 202.98±1.56 | 5.98±0.04 | 9174.69±4591.92 | 53.87±14.05 | 92.28±30.29 | 1993.25±287.88 | 31.5±3 | 401.75±72.16 | 0.15±0 | 1.65±0.04 |
| CK | 1 | 6 | 60.66±9.86 | 30.23±3.85 | 5.99±0.28 | 3.34±2.4 | 4.36±0.66 | 299.59±17.57 | 404.31±66.79 | 176.77±4.05 | 6.34±0.04 | 9531.84±1608.26 | 42.3±15.77 | 73.04±47.71 | 2595.75±228.46 | 40.75±3.3 | 727.25±62.73 | 0.14±0 | 1.59±0.03 |
| RR | 1 | 6 | 64.9±5.39 | 60.19±16.44 | 9.63±1.41 | 5.09±0.6 | 3.99±0.65 | 308.18±17.5 | 648.34±16.27 | 209.57±3.38 | 5.83±0.02 | 17103.42±8680.72 | 61.75±3.58 | 123.17±20.79 | 2286±152.28 | 36±5.89 | 470.5±20.73 | 0.15±0 | 1.6±0.04 |
| CK | 2 | 6 | 52.13±5.22 | 34.93±3.98 | 7.37±0.84 | 2.61±1.53 | 3.69±0.27 | 301.99±8.46 | 362.76±9.43 | 173.78±2.48 | 6.23±0.05 | 13538.5±12141.88 | 51.89±13.33 | 100.74±27.04 | 2689.25±148.87 | 41.5±1.73 | 825.5±69.18 | 0.14±0.01 | 1.83±0.34 |
| RR | 2 | 6 | 72.63±4.03 | 51.06±11.36 | 8.96±0.64 | 3.56±0.79 | 3.38±0.71 | 303.21±14.83 | 665.41±39.87 | 205.34±3.84 | 5.83±0.23 | 19571±8309.56 | 39.78±9.89 | 79.39±44.33 | 2270±254.8 | 32.5±3.7 | 401±37.57 | 0.15±0 | 1.6±0.06 |
| CK | 3 | 6 | 65.13±5 | 34.63±3.61 | 8.17±0.75 | 1.35±0.92 | 5.04±0.67 | 277.58±8.64 | 391.78±13.85 | 178.84±3.22 | 6.04±0.11 | 16304.8±10297.5 | 36.51±1.21 | 54.6±3.84 | 2451.25±174.06 | 41±1.41 | 757.75±116.61 | 0.14±0 | 1.54±0.02 |
| RR | 3 | 6 | 90.86±8.72 | 59.53±8.53 | 10.03±1.16 | 4.46±0.62 | 3.64±0.62 | 286.52±20.48 | 620.21±77.88 | 205.63±6.16 | 5.5±0 | 13049.21±3792.58 | 49.32±11.1 | 99.11±38.73 | 2518±125.86 | 38.75±5.5 | 448±16.83 | 0.15±0.01 | 1.55±0.08 |
| CK | 1 | 12 | 146.3±77.61 | 39.61±9.71 | 9.12±5.73 | 6.1±2.04 | 6.29±3.21 | 335.74±10.78 | 430.7±39.77 | 196.02±8.47 | 5.9±0.03 | 20626.79±5144.59 | 38.16±10.95 | 75.79±51.69 | 2685.5±264.07 | 39.75±3.4 | 822.75±99.42 | 0.14±0.01 | 1.92±0.19 |
| RR | 1 | 12 | 121.6±4.04 | 46.64±10.84 | 6.08±1.2 | 7.19±2.17 | 4.35±0.4 | 403.49±34.55 | 643.04±12.55 | 239.24±3.6 | 5.44±0.01 | 9257.75±6605.37 | 46.48±13.61 | 97.42±55.72 | 2332.75±167.24 | 37.75±2.22 | 425.25±64.06 | 0.15±0.01 | 1.65±0.1 |
| CK | 2 | 12 | 77.83±14.73 | 26.07±5.64 | 4.19±0.83 | 5.05±0.32 | 3.68±0.48 | 359.12±15.59 | 407.11±5.02 | 200.69±0.8 | 5.84±0.01 | 11466.35±9873.58 | 51.34±12.4 | 53.09±40.77 | 2525.5±314.02 | 43±7.75 | 730.75±82.78 | 0.14±0 | 1.57±0.03 |
| RR | 2 | 12 | 115.29±25.36 | 43.03±5.45 | 5.72±0.86 | 10.02±5.81 | 3.6±1.03 | 405.73±34.4 | 607.11±52.04 | 236.94±4.02 | 5.38±0.01 | 9882.35±3619.07 | 54.67±14.47 | 127±45.04 | 2490.75±217.41 | 35.25±5.56 | 410.25±38.81 | 0.15±0.01 | 1.58±0.07 |
| CK | 3 | 12 | 83.99±8.27 | 28.71±8.45 | 4.5±0.59 | 4.41±0.39 | 4.09±0.34 | 362.7±17.16 | 427.63±30.52 | 195.05±2.75 | 5.8±0.03 | 6213.35±2191.33 | 47.49±10.96 | 48.1±10.84 | 2624±85.76 | 37±8.29 | 731±34.9 | 0.14±0.01 | 1.66±0.05 |
| RR | 3 | 12 | 115.12±19.3 | 46.15±8.07 | 7.77±2.48 | 7.52±1.22 | 3.48±0.65 | 391.08±23.82 | 578.54±9.02 | 237.36±4.98 | 5.4±0.01 | 4910.8±2856.35 | 43.38±12.01 | 80.21±43.05 | 1956.5±307.03 | 31.25±6.02 | 383.75±35.19 | 0.15±0 | 1.61±0.05 |

Abbreviations: AK, available potassium; AP, available phosphorus; NO_3_^-^-N, soil nitrate N; NH_4_^+^-N, soil ammonia N; TC, total carbon; TN, total nitrogen; APE, acid phosphatase; BG, β-D-glucosidase; XYL, β-D-xylosidase; LAP, leucine aminopeptidase; PPO, polyphenol oxidase; UE, urease; bac_S, bacterial richness; fun_S, fungal richness; arch_S, archaeal richness; RR, maize residue retention; FT, freeze-thawing; RR*FT intensity, the interaction between RR and FT intensity. *P* values: *P* > 0.05, not significant; *P* < 0.05, significant.

| **Table S2** Permanova analysis examining the effects of residue retention (RR), freeze-thawing (FT) intensity, FT cycles (Cycle), and their interactions on archaeal, prokaryotic and fungal community compositions. | | | | | | |
| --- | --- | --- | --- | --- | --- | --- |
|  | Bacteria | | Fungi | | Archaea | |
|  | *R*^2^ | *P* | *R*^2^ | *P* | *R*^2^ | *P* |
| RR | 0.264 | 0.001 | 0.526 | 0.001 | 0.461 | 0.001 |
| FT intensity | 0.009 | 0.219 | 0.005 | 0.277 | 0.013 | 0.098 |
| Cycle | 0.018 | 0.020 | 0.015 | 0.027 | 0.012 | 0.105 |
| RR*FT intensity | 0.007 | 0.450 | 0.005 | 0.280 | 0.007 | 0.312 |
| RR* Cycle | 0.008 | 0.404 | 0.004 | 0.358 | 0.007 | 0.294 |
| FT intensity*Cycle | 0.008 | 0.354 | 0.008 | 0.132 | 0.003 | 0.721 |
| RR* Cycle*FT intensity | 0.008 | 0.324 | 0.006 | 0.256 | 0.007 | 0.276 |

| Table S3 Mantel test examining the relationships between soil physiochemical variables and archaeal, bacterial, and fungal community compositions. | | | | | | |
| --- | --- | --- | --- | --- | --- | --- |
| Variables | Bacteria | | Archaea | | Fungi | |
|  | *r* | *p* | *r* | *p* | *r* | *p* |
| AK | 0.648 | 0.001 | 0.581 | 0.001 | 0.665 | 0.001 |
| AP | 0.408 | 0.001 | 0.334 | 0.001 | 0.423 | 0.001 |
| PH | 0.162 | 0.001 | 0.106 | 0.004 | 0.087 | 0.022 |
| NH_4_^+^-N | -0.036 | 0.940 | 0.005 | 0.381 | 0.003 | 0.436 |
| NO_3_^-^-N | -0.014 | 0.662 | 0.015 | 0.281 | -0.029 | 0.832 |
| TN | 0.345 | 0.001 | 0.287 | 0.001 | 0.351 | 0.001 |
| TC | -0.022 | 0.637 | -0.085 | 0.995 | 0.095 | 0.044 |

**Abbreviations:** AK, available potassium; AP, available phosphorus; NH_4_^+^-N, ammonium nitrogen; NO_3_^-^-N, nitrate nitrogen; TN, total nitrogen; TC, total carbon.

| **Table S4** Keystone prokaryotic and fungal ASVs in FT1, FT2 and FT3 networks**.** Abbreviations: FT, freeze-thawing; FT1, constant 4 °C; FT2, -4℃/ 4℃ (moderate FT intensity); FT3, -10℃/ 4℃ (severe FT intensity) | | | | | | | | |
| --- | --- | --- | --- | --- | --- | --- | --- | --- |
|  | **asv** | **taxon** | **phylum** | **class** | **order** | **family** | **genus** | **species** |
|  | **ASV27203** | **Bacteria** | **Proteobacteria** | **Alphaproteobacteria** | **Rhizobiales** | **Xanthobacteraceae** | **Pseudolabrys** | **NA** |
|  | **ASV11472** | **Bacteria** | **Acidobacteriota** | **Acidobacteriae** | **Bryobacterales** | **Bryobacteraceae** | **Bryobacter** | **NA** |
| **bac_FT1** | **ASV33355** | **Bacteria** | **Verrucomicrobiota** | **Verrucomicrobiae** | **Chthoniobacterales** | **Terrimicrobiaceae** | **Terrimicrobium** | **NA** |
|  | **ASV1471** | **Bacteria** | **Myxococcota** | **Polyangia** | **Polyangiales** | **BIrii41** | **BIrii41** | **uncultured_bacterium** |
|  | **ASV1368** | **Bacteria** | **Acidobacteriota** | **Holophagae** | **Holophagales** | **Holophagaceae** | **Holophaga** | **Geothrix_sp.** |
|  |  |  |  |  |  |  |  |  |
|  | **ASV6287** | **Bacteria** | **Proteobacteria** | **Alphaproteobacteria** | **Micropepsales** | **Micropepsaceae** | **uncultured** | **metagenome** |
| **bac_FT2** | **ASV21927** | **Bacteria** | **Proteobacteria** | **Alphaproteobacteria** | **Sphingomonadales** | **Sphingomonadaceae** | **Sphingomonas** | **NA** |
|  | **ASV32341** | **Bacteria** | **Proteobacteria** | **Alphaproteobacteria** | **Micropepsales** | **Micropepsaceae** | **uncultured** | **metagenome** |
|  |  |  |  |  |  |  |  |  |
|  | **ASV17301** | **Bacteria** | **Actinobacteriota** | **Thermoleophilia** | **Gaiellales** | **Gaiellaceae** | **Gaiella** | **uncultured_Rubrobacteria** |
| **bac_FT3** | **ASV23714** | **Bacteria** | **Acidobacteriota** | **Acidobacteriae** | **Acidobacteriales** | **uncultured** | **uncultured** | **uncultured_Acidobacteria** |
|  | **ASV6425** | **Bacteria** | **Acidobacteriota** | **Acidobacteriae** | **Solibacterales** | **Solibacteraceae** | **Candidatus_Solibacter** | **bacterium_Ellin7504** |
|  | **ASV18643** | **Bacteria** | **Actinobacteriota** | **Actinobacteria** | **Propionibacteriales** | **Nocardioidaceae** | **Kribbella** | **NA** |
|  |  |  |  |  |  |  |  |  |
|  | **ASV164** | **Fungi** | **unclassified_Fungi** | **unclassified_Fungi** | **unclassified_Fungi** | **unclassified_Fungi** | **unclassified_Fungi** | **NA** |
|  | **ASV275** | **Fungi** | **Ascomycota** | **NA** | **NA** | **NA** | **NA** | **NA** |
| **fun_FT1** | **ASV58** | **Fungi** | **unclassified_Fungi** | **unclassified_Fungi** | **unclassified_Fungi** | **unclassified_Fungi** | **unclassified_Fungi** | **NA** |
|  | **ASV269** | **Fungi** | **Ascomycota** | **Sordariomycetes** | **Trichosphaeriales** | **Trichosphaeriales_family_Incertae_sedis** | **NA** | **NA** |
|  | **ASV395** | **Fungi** | **NA** | **NA** | **NA** | **NA** | **NA** | **NA** |
|  |  |  |  |  |  |  |  |  |
|  | **ASV26** | **Fungi** | **Ascomycota** | **Eurotiomycetes** | **NA** | **NA** | **NA** | **NA** |
| **fun_FT2** | **ASV57** | **Fungi** | **Zygomycota** | **Zygomycota_class_Incertae_sedis** | **Mucorales** | **Mucoraceae** | **Mucor** | **NA** |
|  | **ASV296** | **Fungi** | **unclassified_Fungi** | **unclassified_Fungi** | **unclassified_Fungi** | **unclassified_Fungi** | **unclassified_Fungi** | **NA** |
|  |  |  |  |  |  |  |  |  |
|  | **ASV230** | **Fungi** | **unclassified_Fungi** | **unclassified_Fungi** | **unclassified_Fungi** | **unclassified_Fungi** | **unclassified_Fungi** | **NA** |
|  | **ASV98** | **Fungi** | **Ascomycota** | **Eurotiomycetes** | **Eurotiales** | **Trichocomaceae** | **NA** | **NA** |
|  | **ASV466** | **Fungi** | **Chytridiomycota** | **Chytridiomycetes** | **unclassified_Chytridiomycetes** | **unclassified_Chytridiomycetes** | **unclassified_Chytridiomycetes** | **Chytridiomycetes_sp** |
|  | **ASV73** | **Fungi** | **Ascomycota** | **Sordariomycetes** | **Chaetosphaeriales** | **Chaetosphaeriaceae** | **Chloridium** | **Chloridium_sp** |
|  | **ASV46** | **Fungi** | **Ascomycota** | **Dothideomycetes** | **Pleosporales** | **Venturiaceae** | **NA** | **NA** |
|  | **ASV199** | **Fungi** | **Ascomycota** | **Dothideomycetes** | **Pleosporales** | **Pleosporales_family_Incertae_sedis** | **unclassified_Pleosporales** | **Pleosporales_sp** |
|  | **ASV84** | **Fungi** | **Ascomycota** | **Eurotiomycetes** | **Chaetothyriales** | **unclassified_Chaetothyriales** | **unclassified_Chaetothyriales** | **Chaetothyriales_sp** |
|  | **ASV520** | **Fungi** | **Basidiomycota** | **Agaricomycetes** | **Agaricales** | **Agaricaceae** | **Lycoperdon** | **NA** |
|  | **ASV3** | **Fungi** | **Ascomycota** | **Sordariomycetes** | **Hypocreales** | **Nectriaceae** | **Gibberella** | **Gibberella_intricans** |
| **fun_FT3** | **ASV9** | **Fungi** | **Ascomycota** | **Dothideomycetes** | **Dothideomycetes_order_Incertae_sedis** | **Pseudeurotiaceae** | **Pseudogymnoascus** | **Pseudogymnoascus_roseus** |
|  | **ASV122** | **Fungi** | **Ascomycota** | **Sordariomycetes** | **Coniochaetales** | **Coniochaetaceae** | **Coniochaeta** | **Lecythophora_sp_F31** |
|  | **ASV286** | **Fungi** | **Ascomycota** | **Dothideomycetes** | **NA** | **NA** | **NA** | **NA** |
|  | **ASV35** | **Fungi** | **Ascomycota** | **Ascomycota_class_Incertae_sedis** | **Ascomycota_order_Incertae_sedis** | **Ascomycota_family_Incertae_sedis** | **Clohesyomyces** | **Clohesyomyces_aquaticus** |
|  | **ASV114** | **Fungi** | **Ascomycota** | **Dothideomycetes** | **Dothideomycetes_order_Incertae_sedis** | **Pseudeurotiaceae** | **Pseudeurotium** | **Pseudeurotium_hygrophilum** |
|  | **ASV116** | **Fungi** | **unclassified_Fungi** | **unclassified_Fungi** | **unclassified_Fungi** | **unclassified_Fungi** | **unclassified_Fungi** | **NA** |
|  | **ASV207** | **Fungi** | **unclassified_Fungi** | **unclassified_Fungi** | **unclassified_Fungi** | **unclassified_Fungi** | **unclassified_Fungi** | **NA** |
|  | **ASV30** | **Fungi** | **Ascomycota** | **NA** | **NA** | **NA** | **NA** | **NA** |
|  | **ASV46** | **Fungi** | **Ascomycota** | **Dothideomycetes** | **Pleosporales** | **Venturiaceae** | **NA** | **NA** |
