# Supplementary material for: Maize residue retention shapes soil microbial communities and co-occurrence networks upon freeze-thawing cycles
Source: PeerJ. 2024 Jun 14;12:e17543. doi: 10.7717/peerj.17543 (PMC11182024; doi:10.7717/peerj.17543)
Supplement: Supplemental Information 4 [file peerj-12-17543-s004.docx]

Dear editor,

We are really sorry for the change of authors. Please let me clary the reasons in detail. **Nuriddin Samatov** and **Aziza Usmonkulova** indeed contributed to the current work in the initial submission. In the initial submission, they conducted the data analysis (the effect of residue retention and freezing-thawing on N leaching). However, these results were not included in our manuscript (e.g., the leaching of the N), so we did not include their name in the initial submission. In the second submission, the reviewers suggested that bacterial functional prediction should be included. **Nuriddin Samatov** and **Aziza Usmonkulova** helped us conduct FAPROTAX analysis to predict bacterial function. Also, they helped us revise the language after first decision, which has been commented in the manuscript now. Therefore, given their contributions to the work, we decided to include their name in the submission after careful consideration.
